# Structure of membrane diacylglycerol kinase in lipid bilayers

**DOI:** 10.1038/s42003-021-01802-1

**Published:** 2021-03-05

**Authors:** Jianping Li, Yang Shen, Yanke Chen, Zhengfeng Zhang, Shaojie Ma, Qianfen Wan, Qiong Tong, Clemens Glaubitz, Maili Liu, Jun Yang

**Affiliations:** 1 National Center for Magnetic Resonance in Wuhan, Key Laboratory of Magnetic Resonance in Biological Systems, State Key Laboratory of Magnetic Resonance and Atomic and Molecular Physics, Wuhan Institute of Physics and Mathematics, Innovation Academy for Precision Measurement Science and Technology, Chinese Academy of Sciences, Wuhan, 430071 People’s Republic of China; 2 Laboratory of Chemical Physics, National Institute of Diabetes and Digestive and Kidney Diseases, National Institutes of Health, Bethesda, MD 20892-0520 USA; 3 Institute for Biophysical Chemistry and Centre for Biomolecular Magnetic Resonance, Goethe Universität Frankfurt, 60438 Frankfurt am Main, Germany; 4 Wuhan National Laboratory for Optoelectronics, Huazhong University of Science and Technology, Wuhan, 430074 People’s Republic of China

**Keywords:** Solid-state NMR, Solid-state NMR

## Abstract

Diacylglycerol kinase (DgkA) is a small integral membrane protein, responsible for the ATP-dependent phosphorylation of diacylglycerol to phosphatidic acid. Its structures reported in previous studies, determined in detergent micelles by solution NMR and in monoolein cubic phase by X-ray crystallography, differ significantly. These differences point to the need to validate these detergent-based structures in phospholipid bilayers. Here, we present a well-defined homo-trimeric structure of DgkA in phospholipid bilayers determined by magic angle spinning solid-state NMR (ssNMR) spectroscopy, using an approach combining intra-, inter-molecular paramagnetic relaxation enhancement (PRE)-derived distance restraints and CS-Rosetta calculations. The DgkA structure determined in lipid bilayers is different from the solution NMR structure. In addition, although ssNMR structure of DgkA shows a global folding similar to that determined by X-ray, these two structures differ in monomeric symmetry and dynamics. A comparative analysis of DgkA structures determined in three different detergent/lipid environments provides a meaningful demonstration of the influence of membrane mimetic environments on the structure and dynamics of membrane proteins.

## Abstract (web summary)

Jianping Li et al. present the homo-trimeric structure of the small integral membrane protein diacylglycerol kinase (DgkA) in phospholipid bilayers determined by magic angle spinning solid-state NMR spectroscopy. They compare the structure with structures solved by solution NMR and X-ray crystallography and provide insights into the influence of membrane mimetic environments on membrane proteins.

## Introduction

The structure and function of membrane proteins (MPs) can be influenced by membrane mimetic environments^1,2^. The determination of 3D structure of MPs in native membranes or native-like lipid bilayer environment remains a substantial challenge. Most of the MP structures presently available in the Protein Data Bank (PDB) were resolved by X-ray crystallography and solution NMR in a detergent environment. Although detergent micelles can be used to partially mimic the environment of lipid bilayer, the former differs from the latter in terms of hydrophobic thickness, hydrophobicity, surface curvature, and the number of hydrophilic surfaces. The non-native environment of these membrane mimetics could induce structural perturbations in MPs. Using these perturbed structures for the study of functional models of MPs might lead to misinterpretation of the molecular mechanisms underlying these functions. Therefore, to avoid misinterpretation of insights obtained from detergent-perturbed structures, detergent-based structures of MPs need to be validated by structural data acquired in phospholipid bilayers. However, such bilayer-based structures are scarce because of difficulties associated with the structural characterization of MPs embedded in lipid bilayers.

*Escherichia coli* (*E.coli*) diacylglycerol kinase (DgkA) is an integral trimeric MP (42 kDa) that phosphorylates diacylglycerol within the inner *E. coli* membranes^3^. This protein has served as a model MP for a long time and has been extensively studied in the context of its enzymology^4–7^, folding^8,9^, stability^10^, and structural biology^11–16^. The 3D structures of DgkA have been determined in dodecylphosphocholine (DPC) micelles by solution NMR^16^ and in monoolein cubic phase by X-ray crystallography^15,17^. Notably, the structures determined in these two detergent environments exhibit substantial differences. The structure from solution NMR has a domain-swapping property while the one from X-ray crystallography has not. These differences suggest a possible perturbation of DgkA structure by the detergent environments and highlight the need for the validation of these distinct structures in native-like lipid bilayers.

Magic angle spinning (MAS) solid-state NMR (ssNMR) spectroscopy has emerged as a powerful technique for structure determination of MPs in phospholipid bilayers^18–21^, which are closer to physiological conditions compared to other membrane mimetic environments. In previous studies, Glaubitz and coworkers have demonstrated that DgkA is functionally active in lipid bilayers and its enzyme kinetics can be monitored by real-time ^31^P ssNMR spectra^22^. Further, the global response of DgkA towards substrate binding have been analyzed by 2D and 3D ssNMR spectra, with the cross-protomer contacts being visualized by dynamic nuclear polarization (DNP)-enhanced MAS NMR^23^. Importantly, Yang and coworkers have reported that the secondary structure and membrane topology of DgkA in bilayers of *E. coli* membrane extracts by ssNMR was different from those described earlier in the solution NMR and X-ray structures^24^, suggesting the need for further characterization of the 3D structure of DgkA in lipid bilayers.

However, in our past efforts to determine the structure of DgkA using conventional dipolar-coupling-based 2D ^13^C–^13^C spectra, we failed to obtain long-range distance restraints in numbers sufficient for 3D structure calculations. In this study we aimed to solve 3D structure of DgkA using an approach combining paramagnetic relaxation enhancement (PRE)-derived distance restraints and CS-Rosetta-based^25^ calculations (PRE-CS-Rosetta)^26^. Although PRE-derived distance restraints are advantageous in ssNMR-based structure determination of proteins^27–34^, its use in convenient structure calculation methods was limited by shortcomings associated with large distance uncertainty and high ambiguity. Here, we demonstrate that these shortcomings can be resolved by a CS-Rosetta-based protocol, and a well-defined trimeric DgkA structure can be obtained by using PRE-CS-Rosetta. The monomeric packing architecture of the DgkA structure determined by ssNMR is similar to that of the X-ray structure, but different from that of the solution NMR structure. Comparative analysis of DgkA structures resolved in three different detergent/lipid environments using three structural methodologies provides valuable insights into the influence of membrane mimetic environments on the structure of a multi-span α-helical MP.

## Results

### Difficulties in obtaining long-range distance restraints of DgkA by using conventional approaches

Although ssNMR spectra of DgkA display considerable conformational heterogeneity as indicated by the ^13^C linewidth of 150–200 Hz in the spectra of the U-^13^C, ^15^N-DgkA sample, NMR resonances for 78% of the residues in no-cys-DgkA mutant were assigned based on a set of triple resonance sequential dipolar-coupling-based 3D ssNMR spectra (BMRB ID: 50508). Residues 1–13, 81–88, and 117–121 could not be assigned (Supplementary Data 1), likely due to high mobility.

**Fig. 1:**
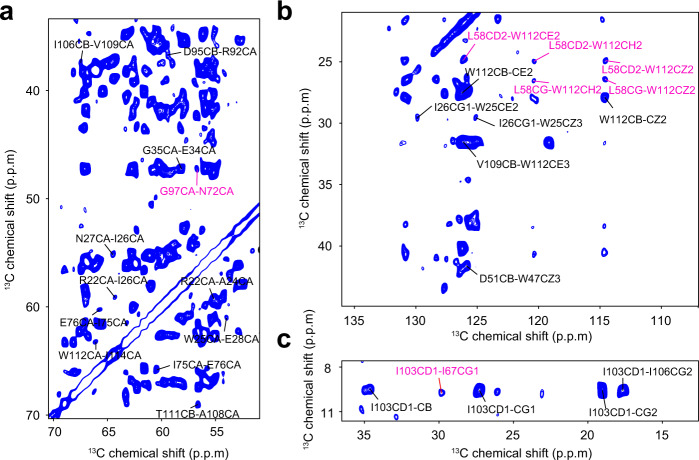
ssNMR spectra of the 13C sparsely labeled DgkA samples. **a, b** Portion of the 2D ^13^C–^13^C DARR spectra recorded on 2-glycerol-^13^C–^15^N-labeled DgkA samples for assigning the ^13^C–^13^C distance restraints. Peaks with an unambiguous assignment are labeled and those corresponding to long-range contacts are highlighted in magenta. **c** Region of the 2D ^13^C–^13^C DARR spectra of 1,3-glycerol-^13^C–^15^N-labeled DgkA sample used for assigning the ^13^C–^13^C distance restraints. Assigned peaks are labeled and those corresponding to long-range contacts are labeled in magenta.

To obtain structural contacts, we recorded a series of 2D ^13^C–^13^C dipolar assisted rotational resonance (DARR) spectra (mixing times from 100 to 1000 ms) of the ^13^C sparsely labeled DgkA samples, expressed in *E. coli* cells using 2/1,3-^13^C-glycerol or 1/2-^13^C-glucose as the sole carbon source (Fig. 1). Further, we conducted a number of 2D ^13^C–^13^C proton-assisted recoupling (PAR) and CHHC experiments using U-^13^C, ^15^N-DgkA samples. However, due to substantial spectral congestion and weak intensity in the dipolar-coupling-based experiments, only 26 long-range distance restraints could be assigned from these 2D ^13^C–^13^C spectra. To distinguish the intra-monomer contacts from inter-monomer ones, we compared 2D ^13^C–^13^C DARR spectra of the diluted samples, prepared by mixing natural abundance monomers with ^13^C-labeled analogs at a 3:1 molar ratio and using an unfolding–refolding approach (see below), with those of undiluted samples^35^. Due to the low sensitivity of diluted samples, we could only unambiguously assign 4 out of 26 long-range constraints as intra-monomer contacts. It is therefore extremely difficult to obtain a well-defined DgkA structure by conventional protein NMR structure determination methods.

### Unfolding and refolding approach for DgkA sample preparations to distinguish intra- and inter-monomer distance restraints

**Fig. 2:**
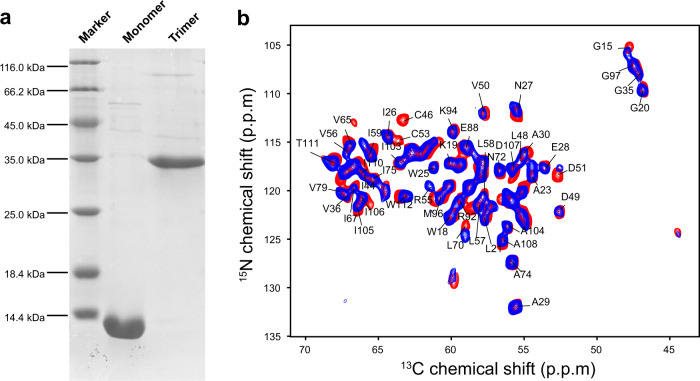
Unfolding and refolding of DgkA. **a** Presence of DgkA monomers formed by unfolding in buffer (8 M urea, 0.2% SDS, 1% formic acid, pH 2.9) and DgkA trimers formed by refolding/reassembling in buffer (8 M urea, 0.2% SDS, 0.08% *E. coli* membrane total extracts, 0.96% OG, pH 7.8) were confirmed by SDS-PAGE. **b** Superposition of the 2D NCA spectrum of no-cys-DgkA undergoing successive unfolding and refolding (blue) with that of wild-type DgkA without unfolding and refolding (red). In no-cys-DgkA, all three cysteine residues (C46, C53, and C113) have been mutated into alanine or isoleucine residues.

To distinguish intra-monomer PREs from inter-monomer ones, individual monomers within a DgkA trimer need to be labeled (spin labeling or isotopic labeling) differently. For differential labeling of individual monomers, the DgkA trimer must be disassembled into monomers, which was achieved by a harsh urea unfolding approach since DgkA forms a highly stable trimer that cannot be dissociated under gentle conditions. In this way, the DgkA trimer was completely disassembled into monomers in buffer (8 M urea, 0.2% SDS, and 1% formic acid at pH 2.9), as shown by SDS-PAGE in Fig. 2a. Under this condition, DgkA was observed to be completely denatured by 2D solution NMR transverse relaxation-optimized spectroscopy (TROSY) experiments (Fig. S1).

The differentially labeled, denatured DgkA monomers were then refolded and reassembled into trimers by adjusting the pH from 2.9 to 7.8 (Fig. S2), adding *E. coli* membrane extracts for reconstitution, and removing urea, SDS and formic acid by dialysis (Fig. S3). After refolding, the oligomeric state and secondary structure of DgkA were examined by SDS-PAGE (Figs. 2a and S4) and CD (Fig. S5), respectively. Comparison of 2D ^15^N–^13^C_α_ (NCA) spectra of refolded and native DgkA samples confirmed that denaturation and reassembling did not perturb the native structure of DgkA (Figs. 2b and S6). In addition, the enzymatic activity of the reassembled DgkA was verified by real-time ^31^P ssNMR spectra (Fig. S7). Therefore, DgkA samples prepared by successive denaturation, refolding, and reconstitution remain correctly folded/reassembled and functional, which allowed us to prepare samples with different labeling schemes to distinguish intra- and inter-monomer PREs.

### Extraction of intra-monomer distance restraints from PRE measurements

**Fig. 3:**
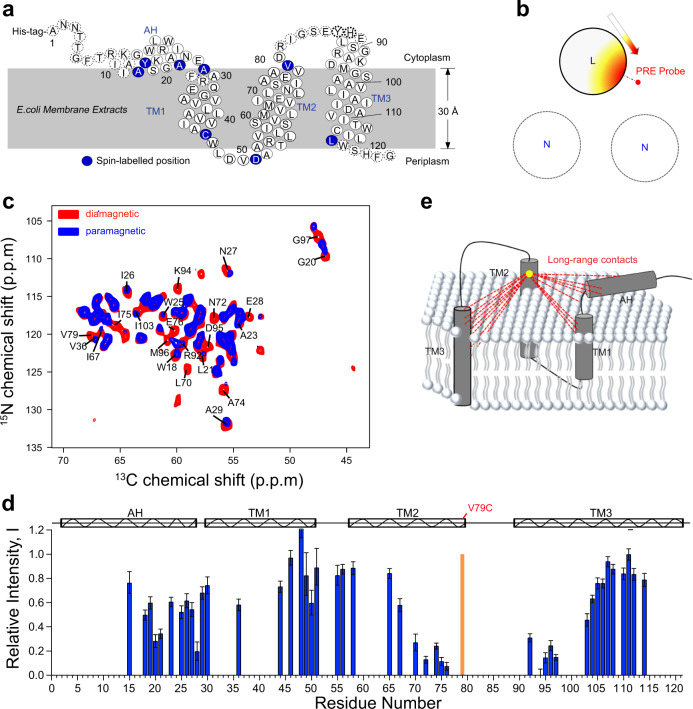
Extraction of intra-monomer distance restraints from PRE measurements using diluted samples. **a** Eight spin-labeled sites (shown as blue balls) for the intra-monomer PRE measurements. **b** Labeling method for observing intra-monomer PREs by spin dilution (see Fig. S11 for more details). “L” and “N” represent uniformly ^13^C,^15^N-labeled and naturally abundant monomers, respectively. **c** Superimposition of the 2D NCA ssNMR spectrum of the paramagnetic sample, ^13^C,^15^N-MTSL-V79C-DgkA/na-wt-DgkA (blue), on that of the diamagnetic cysteine-free sample, ^13^C,^15^N-C46A-C113A-DgkA (red). The peaks with a large attenuation are labeled. **d** Intra-monomer PRE contacts observed for DgkA with a spin-label at the V79C residue. Relative intensity I (given by Eq. 1) calculated from the height of cross-peaks in the NCA spectra of paramagnetic and diamagnetic samples (see panel (**c**)) is plotted for each residue with an observed PRE effect. The noise levels of the corresponding peaks were shown by error bars. The secondary structure of DgkA identified by the ssNMR chemical shifts is shown on the top. The spin-labeling site at V79C is highlighted in orange. **e** Intra-monomer long-range (|i-j| > 5) electron–nuclear distances (dashed lines) obtained from the PREs measured in DgkA mutant with a spin-label site at the V79C (yellow cycle).

To derive the electron–nuclear distance restraints from PRE measurements, eight different residue sites (Fig. 3a) were spin-labeled with the paramagnetic tag 1-oxyl-2,2,5,5-tetramethyl-2,5-dihydropyrrol-3-ylmethyl methane thiosulfonate (MTSL) (Fig. S8). For labeling, cysteine mutations were introduced at positions on the protein surface outside the transmembrane (TM) regions. This enabled full accessibility of MTSL to the labeling sites with a minimal steric hindrance and, at the same time, ensured a lack of interference in the enzymatic activity of DgkA^16^. The completeness of ligation reactions was examined by matrix-assisted laser desorption/ionization time of flight (MALDI-TOF) mass spectrometry, as shown in Fig. S9. The correct folding of all DgkA mutants used in this study was demonstrated by a comparison of their 2D NCA spectra with no-cys ones (Fig. S10).

To collect data on intra-monomer PREs, we diluted U-^13^C,^15^N and paramagnetically labeled monomers with non-labeled (diamagnetic, isotopes at natural abundance) monomers at a molar ratio of 1:4, referred to as the ^13^C,^15^N-MTSL-DgkA/na-DgkA samples. It is noteworthy that dilution of monomers within a trimer was required to suppress the inter-monomer PRE effects (Fig. 3b). An example of the PRE effect is shown in Fig. 3c. The MTSL tag was introduced at residue V79C in TM2 of DgkA, which led to the disappearance or strong attenuation of signals in 2D NCA spectra originating from residues close to the tag. These signals with strong attenuation correspond to residues 18–26 in the amphipathic helix (AH), 28–29 in the TM1 helix, and 91–95 in the TM3 helix (Fig. 3d), indicating the presence of inter-helical contacts between TM2 and AH, TM1, and TM3, respectively (Fig. 3e). To derive a distance restraint from an observed PRE contact, the relative intensity was defined as^27,30^:

1 $${I} = \left( {I_{{\mathrm{para}}}/I_{{\mathrm{dia}}}} \right){\mathrm{/}}\left( {I_{{\mathrm{para}}}/I_{{\mathrm{dia}}}} \right)_{{\mathrm{max}}}$$

where *I*_para_ and *I*_dia_ are the heights of individually resolved peaks in the spectra of paramagnetic and diamagnetic samples, respectively. For the residues at which PRE effect is observed, (*I*_para_/*I*_dia_)_max_ is the maximum value of (*I*_para_/*I*_dia_). We defined the bounds of the PRE restraints using the similar values reported in the previous studies, in which the bounds of attractive and repulsive restraints were determined by empirically analyzing the correlation of attenuated signal intensities (due to PREs) with the corresponding distances in the structures of model protein B1 immunoglobulin-binding domain of protein G (GB1) and *Anabaena* Sensory Rhodopsin (ASR). Accordingly, we defined an attractive restraint with an upper distance limit of 15 Å (between the radical of the tag and the amide proton of the residue) for PRE-affected residues with a relative intensity of *I* < 0.33^19,27,30^, repulsive restraints with a lower limit of 23 Å, and medium restraints with a respective lower and upper limit of 11 and 27 Å^36,37^ for residues with *I* > 0.70 and 0.33 < *I* < 0.70, respectively (Table S1). For all these distance restraints, we used large distance uncertainties, considering residual inter-monomer PRE effects caused by the incomplete dilution, and uncertainties associated with signal intensities in spectra corresponding to different measurements (Figs. S11 and S12). We finally obtained a total of 46 attractive, 113 repulsive, and 64 medium restraints (Table 1), based on data acquired from the eight paramagnetic samples (Table S2). These PRE-derived distance restraints cover nearly all the ordered regions of DgkA.

**Table 1:** PRE distance restraints and refinement statistics for protein structures.

	Protein
*PRE distance restraints (Å)*	
Total PRE restraints	468 × 3
Intra-monomer restraints	223 × 3
*d* < 15	46 × 3
11 < *d* < 27	64 × 3
*d* > 23	113 × 3
Inter-monomer restraints	
*d* < 15	
30 × 3	
11 < *d* < 27	28 × 3
*d* > 23	158 × 3
*d* < 23	29 × 3
*Structure statistics*	
Violations (mean ± s.d.)	
Distance constraints (Å)	NA
Dihedral angle constraints (°)	NA
Max. dihedral angle violation (°)	NA
Max. distance constraint violation (Å)	NA
Deviations from idealized geometry	
Bond lengths (Å)	0.003 ± 0.003
Bond angles (°)	0.673 ± 0.530
Impropers (°)	0.974 ± 1.266
Average pairwise r.m.s. deviation (Å)^a^	
Heavy	3.01 ± 0.22
Backbone	2.03 ± 0.26

^a^Pairwise r.m.s. deviation was calculated between 10 structures, for residues in α-helices, including residues 14–80, 89–116, 135–201, 210–237, 256–322, and 331–358.

### Extraction of inter-monomer distance restraints from PRE measurements

**Fig. 4:**
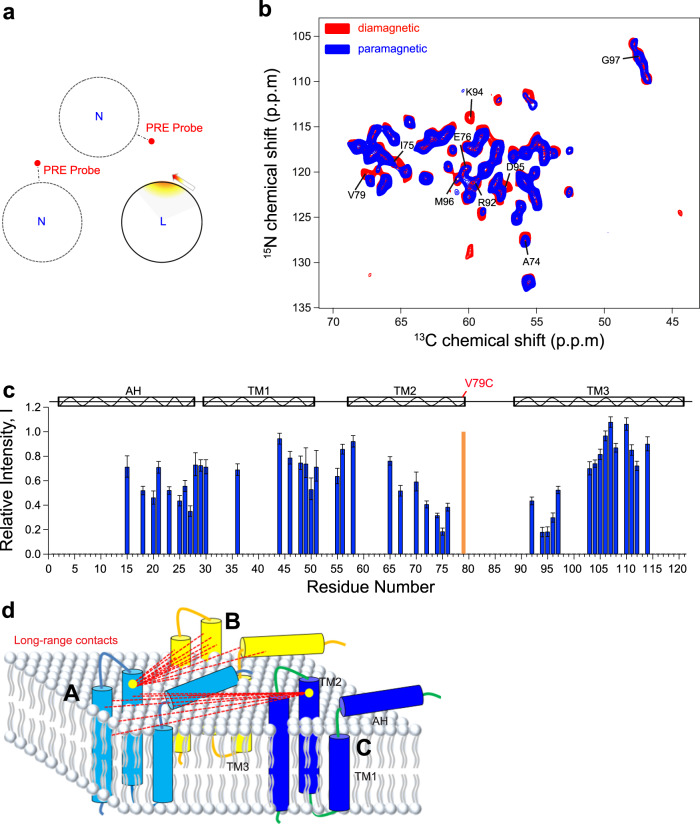
Extraction of inter-monomer distance restraints from PRE measurements. **a** Labeling method for acquiring inter-monomer PREs (see Fig. S12 for more details). “L” and “N” represent uniformly ^13^C,^15^N-labeled and natural abundance monomers, respectively. **b** Superimposition of the 2D NCA spectrum of the paramagnetic sample, na-MTSL-V79C-DgkA/^13^C,^15^N-C46A-C113A-DgkA (blue), on that of the diamagnetic cysteine-free sample, ^13^C,^15^N-C46A-C113A-DgkA (red). The peaks with a large attenuation are labeled. **c** Inter-monomer PRE contacts observed for DgkA with a spin-label at the site V79C. The relative intensity I (given by Eq. 1), calculated from the height of cross-peaks in the NCA spectra **b** of paramagnetic and diamagnetic samples, is plotted for each residue with an observed PRE effect. The noise levels of the corresponding peaks were shown by error bars. The secondary structure of DgkA is shown on the top, with the spin-labeling site of V79C highlighted in orange. **d** Inter-monomer long-range (|i-j| > 5) electron–nuclear distances (dashed lines) obtained from PREs measured in the DgkA mutants with a spin-label introduced at the residue V79C (yellow cycle).

To suppress intra-monomer PRE effects and selectively observe inter-monomer contacts, we prepared three diluted DgkA samples (A24C, 46C, and V79C) by mixing paramagnetically labeled and naturally abundant monomers with U-^13^C,^15^N-labeled ones at a molar ratio of 2:1, referred to as the na-MTSL-DgkA/^13^C,^15^N-DgkA samples (Fig. 4a). We demonstrated an example of measuring the inter-monomer PRE contacts utilizing a paramagnetic tag at the residue V79C. Peak intensities of many residues are significantly attenuated in the 2D NCA spectrum of the paramagnetic sample compared to those in diamagnetic one (Fig. 4b). These residues are located at AH, TM2, and TM3 helices (Fig. 4c), suggesting the occurrence of inter-monomer PRE contacts (Fig. 4d).

However, extraction of unambiguous distance restraints from inter-monomer PRE data is complicated due to the following factors. First, in samples prepared using the dilution strategy mentioned above, residues in the U-^13^C,^15^N-DgkA monomer could be simultaneously affected by the MTSL tags on two other spin-labeled na-MTSL-DgkA monomers. This could lead to an ambiguous and complex scenario, where, two monomers are contributing to the observed PREs. Second, as a consequence of mixing the spin-labeled monomers with the isotopically labeled ones at a molar ratio of 2:1, an ensemble containing four different trimer assemblies characterized by 29.6%, 44.5%, 22.2%, and 3.7% abundance is generated (Fig. S13). Therefore, up to ~1/3 of isotopically labeled monomers in the diluted samples would not experience PRE from the paramagnetic tag (Fig. S13c, d), and leading to 11.1–33.3% contribution to the signals and overlap with those affected by PRE effects. The relative intensity *I* (Eq. 1) affected by inter-monomer PREs, therefore, has a threshold limit of 11.1–33.3% (Fig. 4c), which originated from spins always not affected by PREs (Fig. S13). Considering these two factors, we defined two types of inter-monomer distance restraints at the primary stage of structure calculation: attractive restraints with an upper limit of 23 Å for *I* < 0.70 and repulsive restraints with a lower limit of 23 Å for *I* > 0.70. These two groups of inter-monomer distance restraints were defined ambiguously, i.e., each restraint was assigned to two electron–^1^H pairs between the radical and two amide protons from the same residue in two different monomers (Table S3). These ambiguous inter-monomer restraints, together with intra-monomer ones, were used to calculate a primary structural model by CS-Rosetta, which was then used to resolve the ambiguity in the inter-monomer restraints (see below).

To avoid interference from the residual intensity unaffected by inter-monomer PRE effects in the diluted samples, we prepared three additional undiluted DgkA samples with spin-labels at the sites A14C, Y16C, and A24C, in which all monomers were simultaneously isotope- and spin-labeled. NMR signals of the undiluted samples were, therefore, affected simultaneously by intra- and inter-monomer PRE effects. For example, spin-labeling at A24C (Fig. S14) caused a stronger attenuation of signals of residues (such as R92, K94, D95, M96, G97, and I103; highlighted by dashed rectangles) in the NCA spectra of undiluted samples (Fig. S14d, g) compared to those of diluted ones (Fig. S14f, i); 2D ^13^C–^13^C DARR spectra with a mixing time of 50 ms showed PRE effects consistent with those identified from the NCA spectra (Fig. S15). By comparing the relative intensities of peaks in the spectra of diluted and undiluted samples, intra- and inter-monomer contacts can be distinguished. Two such examples are the peaks corresponding to residues I103 and A104, of which signal intensities were not influenced by intra-monomer PREs (Fig. S14e, h) but reduced due to inter-monomer PREs (Fig. S14f, i).

We used three diluted samples with spin-labels incorporated at positions A24C in TM1, 46C in TM2, and V79C in TM3, for measuring inter-monomer PREs. In addition, we used three undiluted samples with spin-labels at sites A14C, Y16C, and A24C in AH, to observe more PRE contacts between AH helix and other helices, therefore to obtain the orientation of AH helix with great accuracy. All paramagnetic DgkA samples used in this study are listed in Table S2.

### 3D structure determination of DgkA using PRE-CS-Rosetta

To begin with, we performed conventional NMR structure calculations with Crystallography and NMR system (CNS)^38^ using all available structural restraints, including 190 backbone torsion angle restraints from TALOS+^39^, 128 intra-helical hydrogen bonds, and intra-monomer and ambiguous inter-monomer PRE distance restraints. These calculations failed to generate a converged homo-trimeric structure of DgkA, likely due to the ambiguity and large uncertainty in the PRE-derived restraints.

We then employed a CS-Rosetta-based protocol using these semi-quantitative PRE restraints for calculating the homo-trimeric structure of DgkA. In the CS-Rosetta protocol, a Monte Carlo strategy is used to assemble short fragments of known structures in protein data base (PDB) to search for contact and low energy fold of the monomers, in which experimentally measured chemical shifts were employed to improve the structural accuracy of these short fragments. The DgkA trimeric structures were generated by a hybrid symmetric docking protocol using the selected monomer structures. The intra- and inter-monomeric PRE constraints were used in this step to aid the docking and subsequent refinement.

**Fig. 5:**
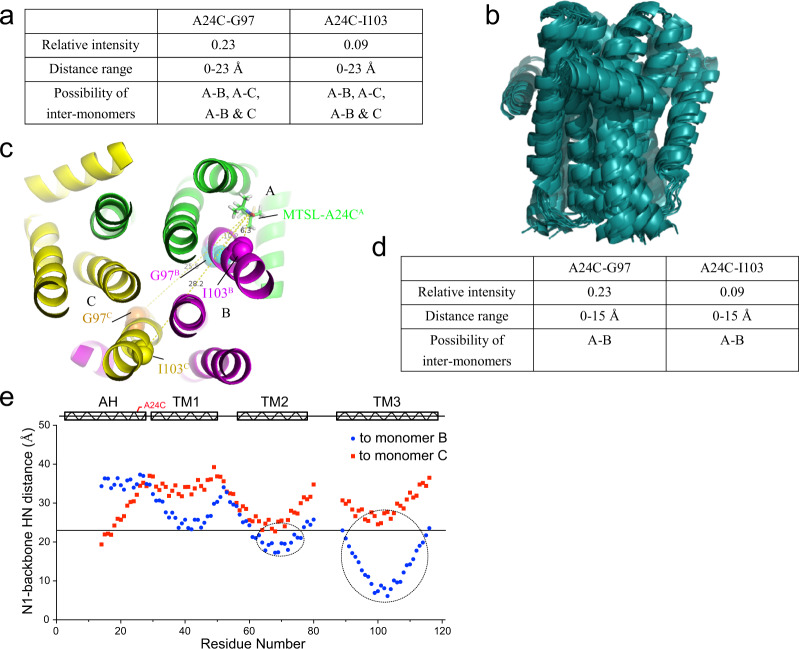
Unambiguous assignment of inter-monomer PRE restraints by an iterative approach using restraints of A24C-G97 and A24C-I103 as examples. **a** Ambiguous inter-monomer PRE restraints of A24C-G97 and A24C-I103 from undiluted A24C MTSL-labeled sample. **b** The 10 lowest energy preliminary structural ensembles calculated by using all the unambiguous intra-monomer PRE restraints and ambiguous inter-monomer restraints in PRE-CS-Rosetta calculation. **c** As measured in the lowest energy structural model, the distances of electron-H^N^ from A24C^A^ to G97^B^ and I103^B^ are 10.2 and 6.3 Å, respectively, while to G97^C^ and I103^C^ are 28.2 and 25.5 Å, respectively. Therefore, the possibilities of inter-monomer PRE effects of A-C and A-B&C monomers can be excluded. **d** Inter-monomer PRE restraints of A24C-G97 and A24C-I103 were unambiguously assigned, and the distance range was reduced from 0–23 Å to 0–15 Å. **e** Distances between the nitroxide radical of the A24C site in monomer A and backbone amide protons in monomers B (blue dot) and C (red dot), as measured in the primary structure model of DgkA. The straight line highlights the distance of 23 Å. The dashed circles represent the relative intensities that are lower than 0.7 in the inter-monomer PRE of the A24C site (see Fig. S14i for more details). The distances between the nitroxide radical at the A24C site in monomer A and backbone amide protons in monomer C (red dot) are almost larger than 23 Å. Hence, those possibilities of inter-monomer PRE effects of A-C and A-B&C monomers could be excluded.

We used a two-step iterative strategy to calculate the structure. In the first step, we obtained an initial homo-trimeric structure of DgkA with a backbone root-mean-square deviation (RMSD) of 2.9 Å (Fig. 5b), by using unambiguous intra-monomer PRE restraints and ambiguous inter-monomer ones. At this step, each inter-monomer PRE restraint is defined to include all possible inter-monomer electron–^1^H contacts, between the nitroxide radical of one monomer, referred to as monomer A, and the amide proton of the affected residue from either or both of the other two monomers (referred to as monomers B and C, Table S3).

In the second step, we utilized the initial homo-trimeric structural model to assign the ambiguous inter-monomer PRE restraints. As an example, we demonstrated the process of assignment of inter-monomer contacts between residues A24C-G97 and A24C-I103 in Fig. 5. Based on the distances between the nitroxide radical at site A24C in monomer A and backbone amide protons in monomers B and C (as estimated from the primary structural model), we could resolve the ambiguity related to these two inter-monomer contacts (Fig. 5e). Based on these assignments, the inter-monomer PRE restraints were now classified into four categories (Table S4). These were: (i) distances unambiguously defined by an upper bound of 15 Å^19,27,30^ if its relative intensity *I* < 0.33; (ii) distances unambiguously defined by respective lower and upper of 11 and 27 Å if 0.33 < *I* < 0.70; (iii) distances unambiguously defined by a lower bound of 23 Å if *I* > 0.70; and (iv) distances (from the diluted samples) ambiguously defined by an upper bound of 23 Å if *I* < 0.70, considering the impact of always unaffected 11.1–33.3% signal intensity. In total, 245 inter-monomer PRE restraints, including 215 unambiguous restraints and 30 attractive restraints with an upper limit of 15 Å, were obtained. All intra- and inter-monomer PRE-derived distance restraints used in the final structure calculation are listed in Table 1.

**Fig. 6:**
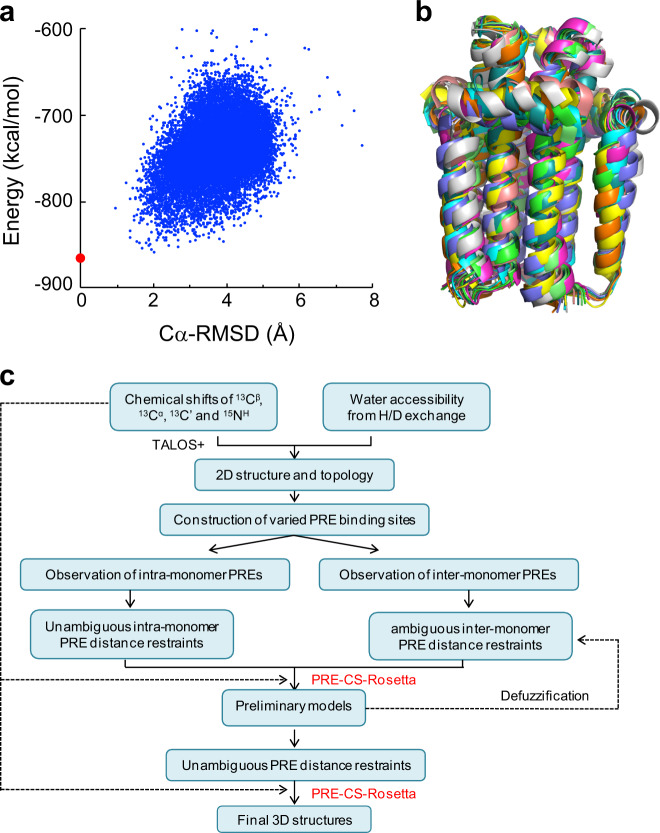
Structure calculations using PRE-CS-Rosetta combined with intra- and inter-monomer distance restraints extracted from PRE measurements. **a** Plot of CS-Rosetta energy score versus C_α_-RMSD relative to the lowest energy structure (red dot) obtained using PRE-CS-Rosetta calculations. **b** An ensemble of 10 structures with the lowest energy from PRE-CS-Rosetta calculations. **c** The workflow of structure determination of membrane proteins by the PRE-CS-Rosetta protocol.

We obtained well-defined homo-trimeric DgkA structures using PRE-CS-Rosetta calculation method and plugging in all available PRE-derived distance restraints. Figure 6a shows the energy profile corresponding to the final CS-Rosetta calculations. As shown in Fig. 6b, the calculated structures are well converged, as demonstrated by a C_α_-RMSD value of 1.8 ± 0.7 Å in the 10 lowest energy structures relative to the lowest energy one. The structures obtained from PRE-CS-Rosetta calculations were validated by ^13^C–^13^C distance restraints assigned from the DARR spectra as mentioned earlier. As shown in Fig. S16 and Table S5, all 26 ^13^C–^13^C distance restraints were consistent with the corresponding distances in the 10 lowest energy structures. It is noteworthy that the converged DgkA homo-trimeric structures were not obtained with a standard CS-Rosetta calculation, i.e., without the use of PRE restraints. This indicates that, regardless of the large uncertainty and high ambiguity, PRE-derived distance restraints are critical to the structure calculation of homo-trimeric DgkA using the CS-Rosetta method. The PRE-CS-Rosetta protocol, as well as the procedure used in this work for the structure determination of DgkA, is summarized in Fig. 6c.

### Comparison of DgkA structure in lipid bilayers with structures reported in detergent environments

**Fig. 7:**
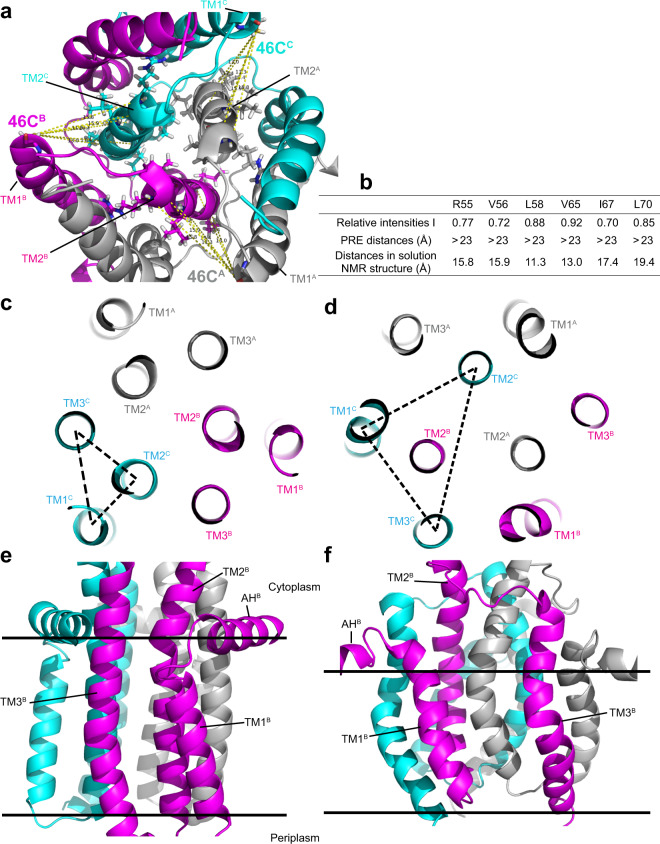
Comparison of the ssNMR DgkA structure reconstituted in *E. coli* membrane total extracts with the one in DPC micelles determined by solution NMR (PDB#: 2KDC). **a** The inter-monomer distance measurements between 46C and residues R55, V56, L58, V65, I67, and L70 in the solution NMR structure (with domain swapping). **b** Comparison of distances from inter-monomer PRE data of the undiluted 46C sample, with those from the solution NMR structure. ssNMR structure of DgkA viewed from the cytoplasm (**c**) and membrane plane (**e**). Solution NMR structure of DgkA viewed from cytoplasm (**d**) and membrane (**f**) planes. Monomers A, B, and C are colored in gray, magenta, and cyan, respectively. In panels (**c** and **d**), TM1, TM2, and TM3 helices of monomer C are connected with a dashed triangle to highlight the differences in monomer packing pattern in the ssNMR and solution NMR DgkA structures. The hydrophobic regions of the ssNMR structure in (**e**) and solution NMR structure in (**f**) were identified based on the analysis of H/D exchange data^24^ and ^19^F NMR data^61^, respectively.

Determination of a well-defined homo-trimeric structure of DgkA reconstituted in *E. coli* membrane extracts allowed us to compare it with other structures available for this MP. Although the reduction of the nativeness of DgkA introduced by mutations, tag linking, unfolding and refolding, low lipid:protein ratio, could not be neglected in this study, the correct structural features and nativeness of the samples used in this study were verified by functional assays. In the trimeric ssNMR structure (Fig. 7e), each monomer contains a short TM helix (TM1), two long TM helices (TM2 and TM3) and an N-terminal AH lying on the surface of membrane. The TM2 helices of each monomer, which are roughly parallel to the bilayer normal, are tightly arranged, forming a core of the three-helix bundle. The AH helices embrace the TM3 helices of adjacent monomers, creating the putative reaction pockets.

The ssNMR structure differs from the solution NMR structure by a C_α_-RMSD of 17.8 Å for the ordered residues of 14–80 and 89–116. Domain swapping, the key structural feature observed in the solution NMR structure, was not observed in the present ssNMR structure (Fig. 7a–d). Figures 7b and S17 show the relative intensities of residues R55, V56, L58, V65, I67, and L70 in the diluted inter-PRE sample tagged with a spin-label at residue 46C. The large relative intensities (*I* > 0.7) of these residues suggest that the corresponding distances are larger than 23 Å. However, the corresponding inter-monomer distances in the solution NMR structure are all less than 20 Å (11–19 Å) due to domain swapping (Fig. 7a). Therefore, we can clearly exclude the possibility of domain swapping in the ssNMR structure based on the above inter-monomer PRE data. In our study, inter- and intra-monomer PREs could be unambiguously distinguished by using samples with different isotopic labeling/dilution schemes, therefore avoiding the use of ambiguous or possibly incorrect constraints for calculating the homo-trimeric structure of DgkA. In addition, some structural features observed in the solution NMR structure, such as disorder in the first 25 residues, an outward curvature of TMs, a large cavity between TMs, and thin hydrophobic thickness, could probably be attributed to the detergent micelles (Fig. 7f). These structural features were not observed in the ssNMR structure (Fig. 7e).

**Fig. 8:**
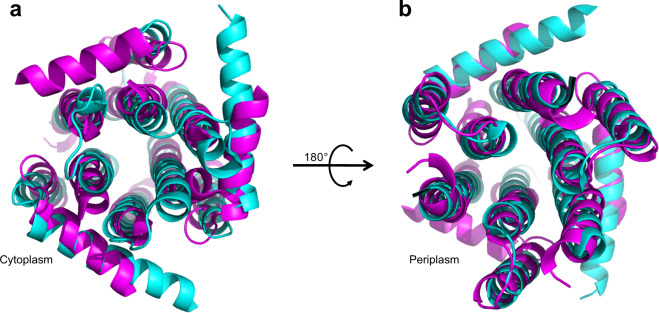
Comparison of the ssNMR DgkA structure reconstituted in *E. coli* membrane total extracts with the one in lipidic cubic phase determined by X-ray crystallography (PDB#: 3ZE4). Superimposition of the ssNMR structure (magenta) to the crystal structure of DgkA (cyan), viewed from cytoplasm (**a**) and periplasm (**b**).

In contrast, the present ssNMR structure shares the general structural features of the X-ray structure (Fig. 8), such as the uniformly cylindrical shape, and the global tight helical packing pattern of the TMs. Analysis of the correlation between the experimentally measured PREs (Fig. S18a, c) and the corresponding distances predicted from the X-ray structure (Fig. S18b, d) showed that all PRE-induced signal attenuations follow the rule of 1/r^6^ dependence on distances, and also demonstrated the similarity of the structure derived from PREs measurements and the X-ray structure. Overall, the ssNMR structure exhibits a C_α_-RMSD of 2.4 Å for the ordered residues (14–80 and 89–116 for monomers A, and B and 28–80 and 89–116 for monomer C) relative to X-ray structure. The similarity between ssNMR and X-ray structure is also consistent with the results from a study of oriented sample ssNMR^40^. However, crystallization requires the packing of protein molecules, which could induce non-native conformational states and dynamics. In the case of DgkA, small asymmetries were found between the protomers and the nucleotide, and lipid substrates were found only in one of the three binding pockets, which could be expected to influence the enzymatic mechanism for the native protein^17^. However, a previous ssNMR study has shown that the apo-, nucleotiode-, and lipid substrate-bound states of DgkA, saturated under conditions similar to those required for crystallization, show homogeneous, well-resolved 2D and 3D spectra without peak multiplets, indicating a symmetric structure^23^. Furthermore, our ssNMR structure exhibits differences to X-ray structure in terms of the dynamics of the AH and the loop between TM2 and TM3. Residues 1–13 of the AH are shown with high dynamics, evidenced by the facts of the absence of their signals in dipolar-based spectra (such as 2D NCA) of DgkA samples in *E. coli* membranes and the observation of their J-coupling-based signals when DgkA samples were prepared in DMPC/PG lipids^41^. Similarly, residues 80–88 in the loop linking TM2 and TM3 are of high flexibility, indicated by the weak sensitivities of their peaks in dipolar-based spectra. While in the X-ray structure, the residues in these two segments are only shown with slightly higher B-factors relative to the residues in the other regions of the protein. Dynamics are essential to the functional activities of the MPs. Both AH and the loop linking TM2 and TM3 are the indispensable components of the binding pockets of reactants. The dynamics of these two segments are likely related to the diffusion of the reactants into the pockets and the release of the product away from the reaction sites.

## Discussion

Although there are a couple of high-resolution ssNMR structures reported for MPs, it does not represent a routine approach for structure determination of MPs. MPs with a known ssNMR structure are limited to the ones with high conformational homogeneity and rigidity of TM domains. DgkA does not belong to this class of MPs. The ^13^C linewidth, indicative of the conformational homogeneity, of the U-^13^C,^15^N DgkA sample in our work is 150–200 Hz, which is much broader than a typical ^13^C linewidth of <100 Hz as observed in other studies such as the one on *Anabaena* Sensory Rhodopsin^19^. The moderate resolution of the ssNMR spectra of DgkA limits the acquisition of unambiguous long-range distance restraints sufficient for high-resolution structure determination, even when a couple of ^13^C sparsely labeled samples were used. The present study demonstrates the feasibility of MP structure determination with the moderate resolution of ssNMR spectra using a CS-Rosetta-based protocol and the sole input of PRE-derived distance restraints.

The use of the PRE-derived distance restraints in ssNMR-based structure determination has obvious advantages. Since PRE effects are identified by comparing site-specific attenuation of signals, PRE restraints can be straightforwardly assigned from simple 2D spectra such as NCA or DARR with a short mixing time. Long-range structural restraints of up to ~20 Å, which provide critical structural information regarding protein fold and oligomerization, could be derived from PREs due to the high gyromagnetic ratio of the incorporated radical electron. The nitroxide radical of MTSL can be easily introduced into different sites of MPs, therefore covering complementary fragments of MPs required for the extraction of a sufficiently large number of PRE-derived restraints.

Regardless of these advantages, PRE-derived distance restraints are generally of low precision and not suitable for standalone use in standard NMR methodologies for calculating high-resolution protein structures. Instead, CS-Rosetta, an alternative structure calculation method, could be utilized to process the structural information provided by PREs in a better way to calculate the structures at high resolution. Consistent structure generation from NMR chemical shift data has recently become feasible for proteins with sizes of up to 370 residues^42–44^ and protein complexes with a higher-order symmetry^45,46^, and the calculated structures are of a quality comparable to those obtained with the standard NMR protocol. The structural information encoded in the NMR data, such as the chemical shifts, RDCs, and sparse NOEs, can be better utilized by combining Rosetta’s sampling and scoring capabilities for protein folding to generate high-resolution protein structures from the available sparse NMR data. In this work, a multi-step PRE-CS-Rosetta protocol was designed to utilize PRE-derived distance restraints for the determination of the well-defined homo-trimeric structure of DgkA. The potential of the PRE-CS-Rosetta protocol to be applicable in ssNMR-based MP structure determinations, even for MPs with considerable flexibility and heterogeneity, is demonstrated here. Compared to similar methods, such as rosettaEPR^47^ and FRET-Rosetta^48^, a larger number of distance restraints can be obtained by introducing a paramagnetic probe in ssNMR PRE method, fascinating the calculation of the highly converged structure. It should be noted that the used low lipid:protein ratio of approximately 25:1 (molar ratio) may induce contacts between different trimers, which on the one hand could contribute to the moderate spectral quality via heterogeneous interactions. On the other hand, inter-trimer PRE effects cannot fully be excluded in the analysis. However, we find that increasing the lipid:protein ratio to 100:1 results in insufficient sensitivity for extraction of PRE, denoting limitations of the used approach.

Structures of helical MPs, especially small helical MPs like DgkA, are sensitive to the membrane mimetic environment due to the following factors. First, helical MPs have high hydrophobic content in the TM domains, hence inter-helical electrostatic interactions in the MPs are weaker compared to that in water-soluble proteins. Second, the inter-helical surface in small helical MPs is smaller than the interacted surface between membrane and protein. At present, solution NMR-based analysis of MP structure is carried out predominantly for MPs in a micellar detergent environment. The non-native features of detergent micelles, such as low hydrophobicity, single hydrophilic surface, and a high degree of curvature, may cause perturbations in the structures of MPs for which structures are being determined.

The perturbation of the native structure of DgkA by DPC micelles in the solution NMR structure could be checked by comparing it with the structure determined by ssNMR in lipid bilayers. The disorder of the first 25 residues in the solution NMR structure, compared to the regular secondary structure of residues 14–25 of the surface AH observed in the ssNMR structure, suggests that the AH points away from the micelles and extends into the bulk solvent. The disorder of AH likely originates from the small size and high degree of curvature of DPC micelles. In addition, the outward curvature of the TM helices in the solution NMR structure, compared to the nearly vertical TM helices in the ssNMR structure, could be attributed to the curved hydrophilic surface of detergent micelles, which exerts an attractive force on hydrophilic residues of TM helices and perturbs their native structure.

Lipidic cubic phase (LCP) has been proven to be a better membrane mimetic for MP crystallization than detergent micelles, especially for small helical MPs^49,50^. LCP composed of monoolein, used for the structure determination of DgkA, has bicontinuous hydrophilic surfaces and highly hydrophobic interstices. These native-like features of LCP make the X-ray structure of DgkA less perturbed by monoolein detergent. Indeed, compared to the structure characterized in lipid bilayers, the X-ray structure of DgkA exhibits typical native-like structural characteristics, such as uniformly cylindrical shape, the global tight helical packing, according to the criteria proposed by Zhou and Cross^1^.

The structures of a number of small helical MPs, including that of KvAP, Smr, phospholamban, KCNE1, and influenza virus AM2, have been reported to be drastically influenced by the membrane mimetic environment^51–55^. In particular, the influence of membrane mimetic environments on the structures of single TM influenza virus AM2 has been extensively analyzed by comparing its 3D structures in three different hydrophobic environments^55^. The present study provides the other example of comparative analysis of 3D structures of a multi-span α-helical MP determined in three different detergent/lipid environments. The comparison provides valuable insights into the influence of membrane mimetic environments on MP structures.

In conclusion, we determined the well-defined homo-trimeric structure of DgkA in bilayers of *E. coli* membrane extracts by ssNMR using an approach combining PRE distance restraints to CS-Rosetta calculations. Domain swapping, a key feature observed in the solution NMR structure but not in those determined by X-ray diffraction, was not observed in the present ssNMR structure. The similarity in global folding and helical packing between structures determined by ssNMR and X-ray validates the X-ray structure analyzed in monoolein cubic phase. Comparative analysis of 3D structures of DgkA determined in three different detergent/lipid environments using three distinct structural methodologies provides insights into the influence of membrane mimetic environment on the structure of a multi-span α-helical MP.

## Methods

### Expression and purification of DgkA

Seven single cysteine mutants A14C, Y16C, A24C, A29C, D51C, V79C, and L116C of DgkA were constructed using the quick-change site-directed mutagenesis kit. The plasmid of no-cys-DgkA (Fig. S19), psD005, containing a N-terminus 6-histidine-tag, was expressed in *E. coli* BL21(DE3) cells. Natural abundance and U-^13^C,^15^N-labeled DgkA analogs were expressed in LB or M9 minimal medium containing 1 g/L ^15^NH_4_Cl and 2 g/L ^13^C-glucose, respectively. The n-dodecyl-N, N-dimethylamine-N-oxide (LDAO) detergent was added to the cell lysate solution with a final concentration of 1% (M/V). DgkA was extracted from the membrane by tumbling at 277 K for 2 h. After centrifugation at 48,384*g* for 16 min, the supernatant was mixed with equilibrated Ni-IDA resin for 2 h by tumbling at 277 K. Then the mixture was flown through a 10 mL column and Ni-IDA resin bound with DgkA was loaded into the column.

The loaded column was sequentially washed in four steps as previously reported^56^. In the first step, the resin was washed using 5 column volumes of the 1^st^ washing buffer (20 mM imidazole, 0.2 % LDAO, 10 mM DTT, 40 mM HEPES, 300 mM NaCl, pH = 7.8). In the second step, 5 column volumes of the 2^nd^ washing buffer (0.2% LDAO, 10 mM DTT, 40 mM HEPES, 300 mM NaCl, pH = 7.8) was flown through the resin. In the third step, the column was washed using 2 column volumes of the 3^rd^ washing buffer (0.1% LDAO, 10 mM DTT, 20 mM HEPES, 240 mM NaCl, pH = 7.8). The fourth step, a key step for refolding the DgkA trimer, consists of washing the resin with 70 column volumes of the 4^th^ washing buffer (8 M Urea, 150 mM NaCl, 10 mM DTT). DgkA was finally eluted using 20 column volumes of the elution buffer (8 M urea, 0.2% SDS, 1% formic acid, 150 mM NaCl, 10 mM DTT, pH = 2.9). The purity of the eluted DgkA monomer was checked by SDS-PAGE (Figs. 2a and S2). The final yield of U-^13^C,^15^N DgkA was ~30 mg per 1 L M9 minimal medium.

### Reassembling of the DgkA trimer

The DgkA monomers were reassembled into trimers by changing the denaturation conditions in a stepwise manner. After changing the pH from 2.9 to 7.8, a small amount of DgkA dimer and trimer was formed (Fig. S2). The resulting protein solution was first pipetted into a suspension of *E. coli* membrane total extracts, which was previously dissolved in *n*-octyl-β-*D*-glucoside (OG) detergent followed by incubation at 37 °C for 13 h to induce refolding of the DgkA trimer. The final ratio of DgkA, *E. coli* membrane total extracts, and OG in the mixture was 1:1.25:15 (w/w/w). For preparing the intra-molecular PRE samples, U-^13^C,^15^N-labeled single cysteine containing DgkA monomers were mixed with unlabeled cysteine-free DgkA monomers in a molar ratio of 1:4 prior to reassembling. The inter-molecular PRE samples were prepared by mixing natural abundance, single cysteine containing DgkA monomers with U-^13^C,^15^N-labeled cysteine-free DgkA monomers in a molar ratio of 1:2 prior to reassembling.

### Ligation of MTSL to DgkA mutants

The ligation reaction of MTSL to DgkA mutants is outlined in Fig. S8. The reassembled DgkA was reduced by 20 mM DTT for 2 h at room temperature. DTT was subsequently removed by dialysis against the dialysis buffer (25 mM NaPi, pH 6.6, 10 mM KCl, 0.2 mM EDTA) in a 10 kDa cut-off dialysis bag at 18 °C. A 10-fold molar excess of MTSL (Toronto Research Chemicals Inc.) was then pipetted into the protein solution. The mixture of proteins and MTSL was shaken at 220 rpm for 2 h at 37 °C, followed by mixing for 12 h with tumbling at 30 °C. Another 10-fold molar excess of MTSL was added to the protein solution. The completeness of incorporation of MTSL into the DgkA mutants was verified by MALDI-TOF mass spectrometry (Fig. S9). Free MTSL was removed by dialysis during the reconstitution of DgkA.

### Preparation of spin-labeled DgkA proteoliposomes

The spin-labeled DgkA solution was packed into a dialysis bag with 10 kDa cut-off and dialyzed for 15 days at 18 °C to ensure complete removal of detergent, urea, and free MTSL (Fig. S3). The dialysis buffer was changed twice every day. The proteoliposomes were collected by ultracentrifugation at 508,735*g* for 2 h using 90 Ti rotor (Beckman Instrument, Fullerton, CA). The wet proteoliposomes were lyophilized and then packed into thin-wall 3.2 mm rotors (Bruker), followed by the adding of 30% ddH_2_O.

### Solid-state NMR spectroscopy

In this work, 3D ssNMR NCACX, NCOCX, and CONCA experiments were performed for chemical shift assignments; 2D ssNMR NCA and DARR experiments were used to acquire PRE distance restraints. All NMR experiments were performed at 272 K on an 800 MHz Bruker Avance III spectrometer equipped with a 3.2 mm E-free ^1^H–^13^C–^15^N probe, at a MAS frequency of 10500 ± 5 Hz. The chemical shifts of ^13^C and ^15^N were indirectly calibrated by using the adamantane as an external referencing standard (40.48 ppm for the downfield carbon)^57^.

The typical 90° pulse lengths of ^1^H, ^13^C, and ^15^N nuclei were 3.1, 4.2, and 6.7 μs, respectively. Radio frequency (RF) of 50 or 40 kHz was used during ^1^H–^13^C/^15^N cross-polarization for ^13^C or ^15^N nuclei with a linear amplitude ramp on ^13^C/^15^N (80–100%). The band-selective polarization was transferred from ^15^N to ^13^C by 4 ms SPECIFIC-CP^58^ with a linear amplitude ramp on ^13^C (93–100%) and RF field power of ~15.8, ~26.3, and 80.6 kHz on ^15^N, ^13^C, and ^1^H, respectively. The ^1^H RF power was set to 65 kHz for decoupling during the acquisition and evolution periods in the 2D/3D experiments. All the multidimensional NMR spectra were processed in NMRPipe^59^ and analyzed using Sparky.

### Analysis of PRE data

Only well-resolved NMR peaks were used to extract PRE distance restraints. To derive a distance restraint from observed PRE data, the relative intensity was defined as shown in Eq. 1. Considering the systematic error in peak intensity, only reliable PRE data were selected to provide distance restraints by following two rules. First, only those residues whose adjacent residues exhibited a similar signal attenuation trend could be used as PRE distance restraints. For example, at the spin-labeled site of L116C, all residues from 44–55 had relative intensities below 0.33, while residue V56 showed a relative intensity of 0.67. According to the above rule, data from V56 would not be selected as a constraint. Second, similar signal attenuation trends should be observed in different nuclei for the same residue in 2D NCA and DARR spectra, otherwise, it would be excluded. For example, except C_γ_ nucleus, C_α_, C_β_, and C_δ_ nuclei of residue L58 had relative intensities greater than 0.7 in the spin-labeled site of V79C. Therefore, PRE data from the C_γ_ nucleus would not be considered.

### Structure calculation of DgkA trimer by PRE-CS-Rosetta

DgkA trimer structures were calculated through a hybrid CS-Rosetta-based structure calculation protocol. This protocol, referred as PRE-CS-Rosetta, uses backbone NMR chemical shifts and PRE distance constraints as experimentally derived input parameters, and generates a well-defined full atom structure of homo-oligomers in a membrane environment. The workflow of the PRE-CS-Rosetta protocol used in this work includes the following steps. First, standard CS-RosettaCM protocol 42 was used to generate monomer structures based on backbone (^15^N, ^13^C_α_, ^13^C_β_ and ^13^C’) chemical shifts. Here, 10,000 models were generated, out of which the lowest energy structures were selected for further analysis. Second, a Rosetta fixed backbone design protocol was used to replace A14, Y16, A24, A29, C46, D51, V79, and L116 with a MSTL-tagged cysteine residue in the selected lowest energy monomer structure. Third, a hybrid symmetric docking protocol^45^ was then adapted to generate the DgkA trimeric structure by symmetrically docking the selected monomer structures in a specific membrane mimetic environment^60^. The experimentally derived PRE constraints were used in this step to aid the docking and subsequent refinement. Here, 20,000 trimer models were generated and the 10 lowest energy structures were selected as the final trimer structures.

### Reporting summary

Further information on research design is available in the Nature Research Reporting Summary linked to this article.

## Supplementary information

Supplementary information.

Description of Additional Supplementary Files.

Supplementary Data 1.

Supplementary Data 2.

Reporting summary.

## Data Availability

The datasets generated during and/or analyzed during the current study are available from the corresponding author on request; 3D structures of DgkA from PRE-CS-Rosetta calculations have been deposited in the Protein Data Bank under accession code 7DVM. Source data for Figs. 3d and 4c are provided in Supplementary Data 2.
