# Supplementary material for: Structure of membrane diacylglycerol kinase in lipid bilayers
Source: Commun Biol. 2021 Mar 5;4:282. doi: 10.1038/s42003-021-01802-1 (PMC7935881; doi:10.1038/s42003-021-01802-1)
Supplement: Supplementary file 1 — Supplementary information. [file 42003_2021_1802_MOESM1_ESM.pdf]

Supplementary Information

Structure of membrane diacylglycerol kinase in lipid bilayers

Jianping Li[1], Yang Shen[2], Yanke Chen[1], Zhengfeng Zhang[1], Shaojie Ma[1], Qianfen Wan[1], Qiong Tong[1], Clemens Glaubitz[3], Maili Liu[1], Jun Yang[1,4]

1. National Center for Magnetic Resonance in Wuhan, Key Laboratory of Magnetic Resonance in Biological Systems, State Key Laboratory of Magnetic Resonance and Atomic and Molecular Physics, Wuhan Institute of Physics and Mathematics, Innovation Academy for Precision Measurement Science and Technology, Chinese Academy of Sciences, Wuhan, 430071, P. R. China.

2. Laboratory of Chemical Physics, National Institute of Diabetes and Digestive and Kidney Diseases, National Institutes of Health, Bethesda, Maryland 20892-0520, United States

3. Institute for Biophysical Chemistry and Centre for Biomolecular Magnetic Resonance, Goethe Universität Frankfurt, Max-von-Laue Strasse 9, 60438 Frankfurt am Main, Germany

4. Wuhan National Laboratory for Optoelectronics, Huazhong University of Science and Technology, Wuhan, 430074, P. R. China.

This file includes:
Figures S1- S19
Tables S1- S5

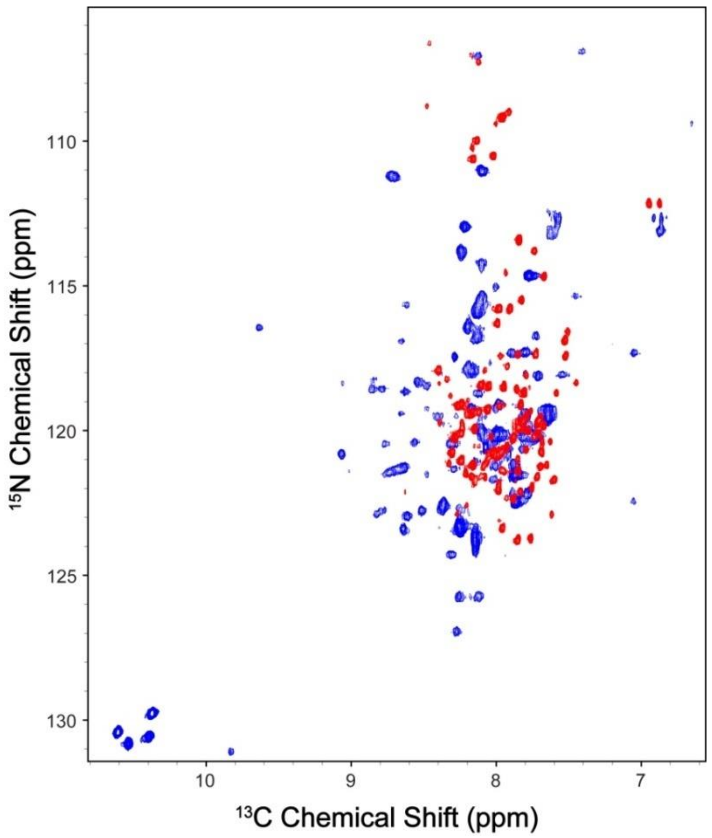

Figure S1. 600 MHz 2D ^{1}H-^{15}N TROSY spectra of DgkA in 0.5% DPC and 250 mM imidazole at pH 6.5 (blue) or in 0.2% SDS, 8 M urea and 1% formic acid at pH = 2.9 (red). Both spectra were recorded at 318 K.

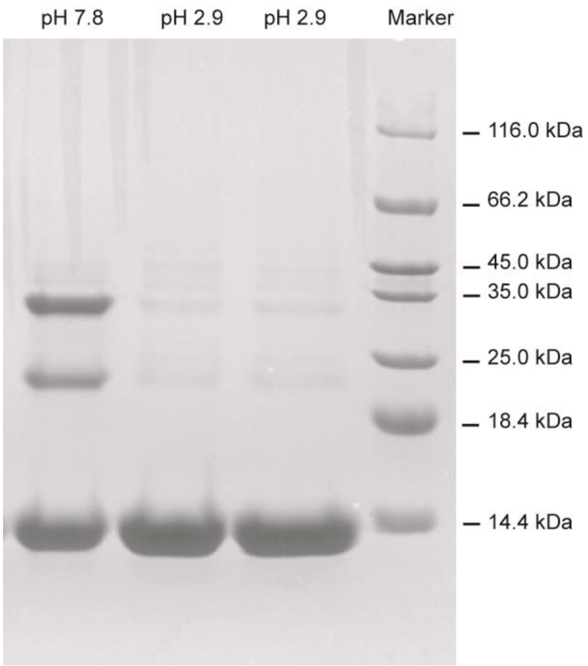

Figure S2. Dissociation of the trimeric no-cys-DgkA mutant denatured in 0.2% SDS, 8 M urea, and 1% formic acid at pH 2.9 or 7.8 as demonstrated by SDS-PAGE gel.

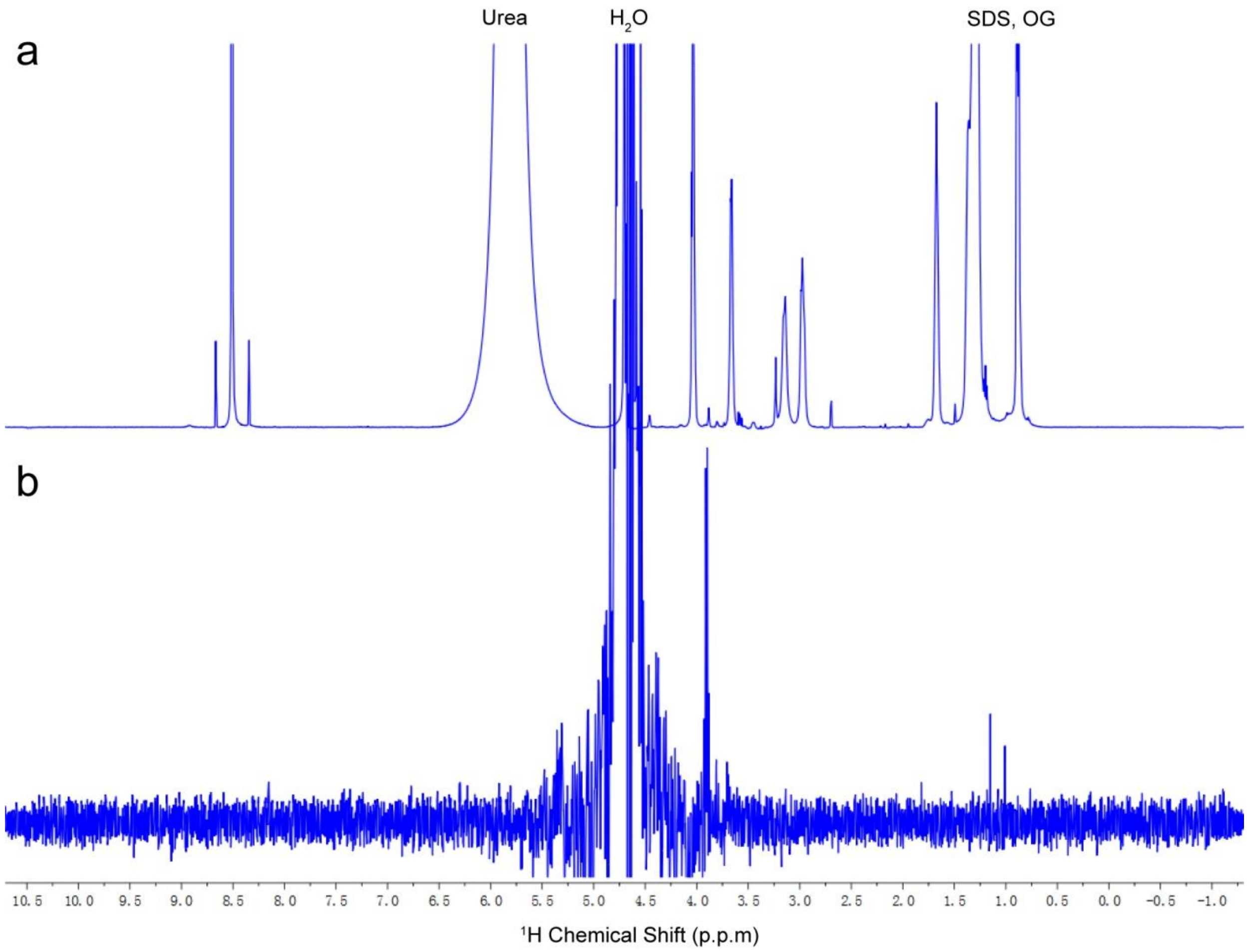

Figure S3. The presence of detergent in the DgkA-*E. coli* lipids-OG-SDS-urea system was monitored by ^{1}H NMR spectra before (a) and after 15 days of dialysis (b). The ^{1}H NMR spectra demonstrated that the SDS and OG detergents could be thoroughly removed after 15 days of dialysis.

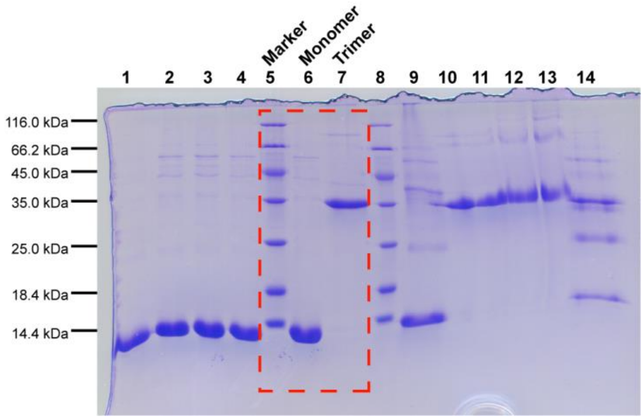

Figure S4. Full, uncropped gel image in **Fig. 2a**. The region labeled with a dashed box was displayed in Fig.2a in the main text. Lane 5 and 8, Marker; Lane 1-4 and 6, A29C_DgkA monomers formed by unfolding in buffer (8M urea, 0.2% SDS, 1% formic acid, pH 2.9); Lane 7, DgkA trimers formed by refolding/reassembling in buffer (8M urea, 0.2% SDS, 0.08% *E.coli* membrane extracts, 0.96% OG, pH 7.8); Lane 9, H87C_DgkA monomers formed by unfolding in buffer (8M urea, 0.2% SDS, 1% formic acid, pH 2.9); Lane 10-13, H87C_DgkA trimers formed by refolding/reassembling in buffer (8M urea, 0.2% SDS, 0.08% *E.coli* membrane extracts, 0.96% OG, pH 7.8); Lane 14, H87C_DgkA trimers formed by refolding/reassembling in buffer without E.coli membrane extracts (8M urea, 0.2% SDS, 0.96% OG, pH 7.8).

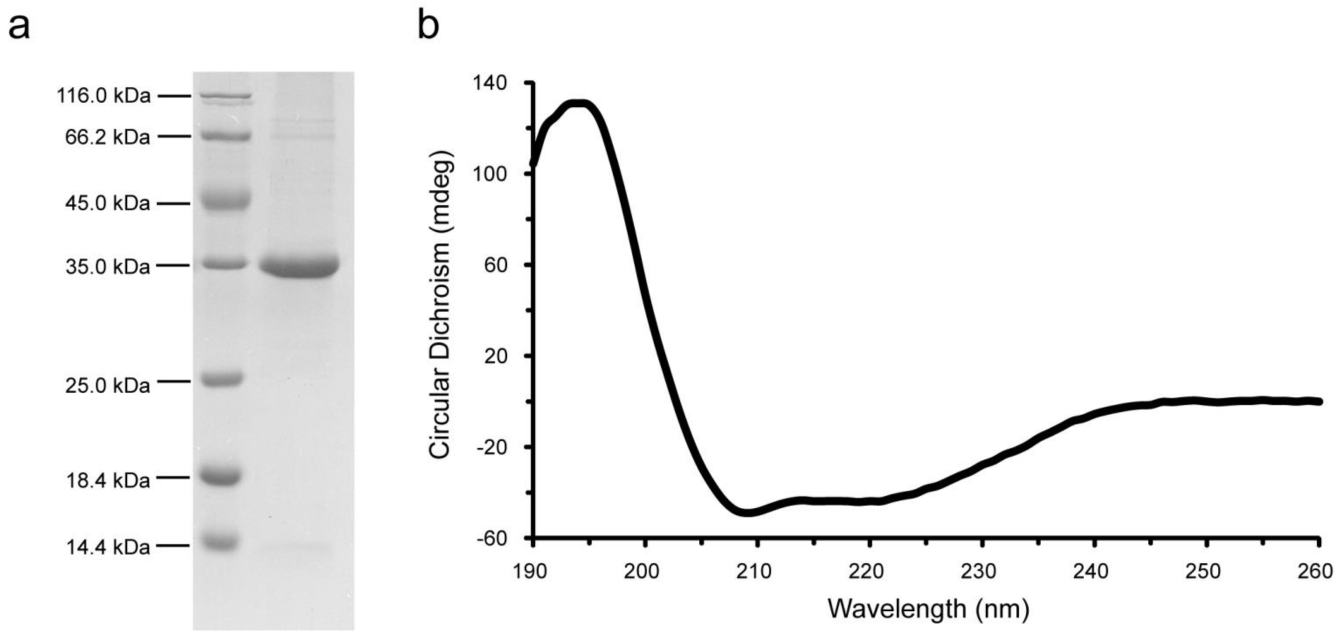

Figure S5. Oligomeric state and secondary structure of no-cys-DgkA in bilayers of *E.coli* membrane extracts detected by (a) SDS-PAGE and (b) circular dichroism spectrum, respectively.

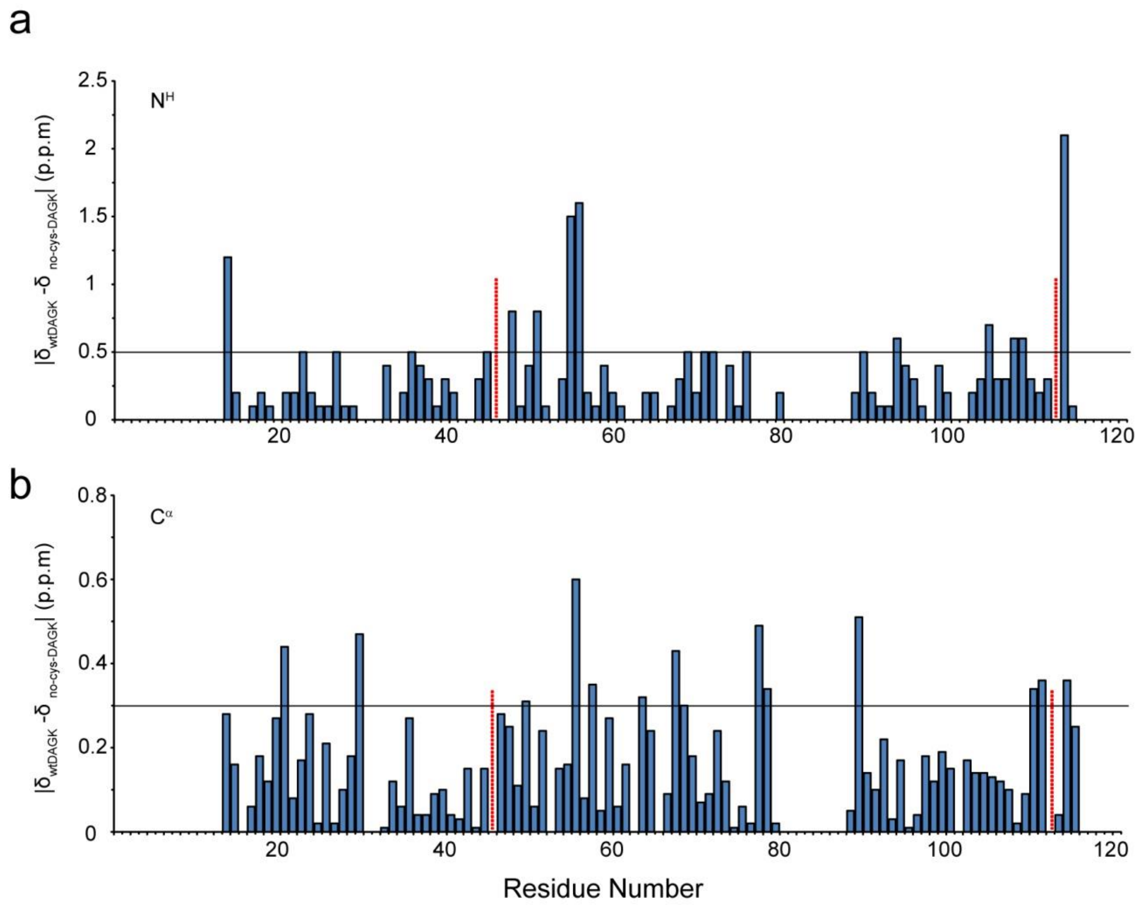

Figure S6. Quantitative comparison of ssNMR chemical shifts from cysteine-free DgkA undergoing unfolding and refolding with those from DgkA without unfolding and refolding. Absolute chemical shift differences of (a) N^H and (b) C_α are shown. The mutant sites of C46A and C113A were indicated by vertical dashed lines in red. Chemical shift differences of residues without assignment were set to zero. As the horizontal black lines show, an overwhelming majority of absolute differences of chemical shifts were within 0.5 and 0.3 ppm for N^H and C_α, respectively.

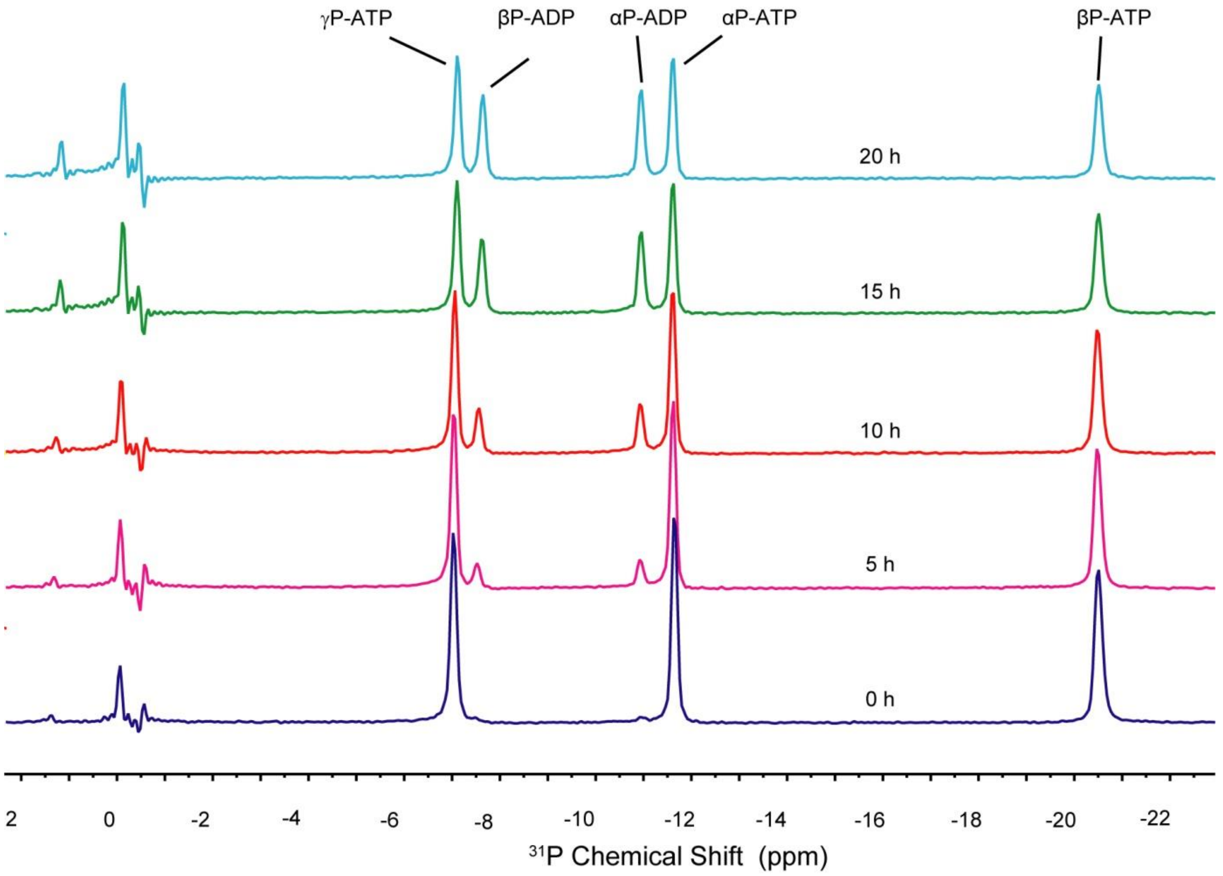

Figure S7. Real-time 1D MAS NMR ^{31}P spectra to monitor the presence of reactant and product during the enzymatic reactions of reassembled DgkA, which has undergone unfolding and refolding, reconstituted into *E. coli* membrane extracts. The increase in the product (ADP), along with a corresponding decrease in the reactant (ATP) with reaction time indicates that the reassembled DgkA trimer reserved its enzymatic activity.

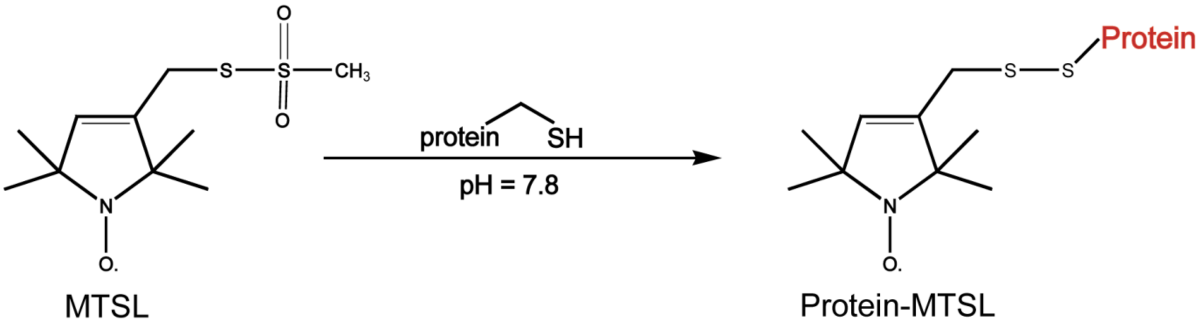

Figure S8. Reaction scheme for incorporation of MTSL at cysteine side chains of DgkA at pH 7.8.

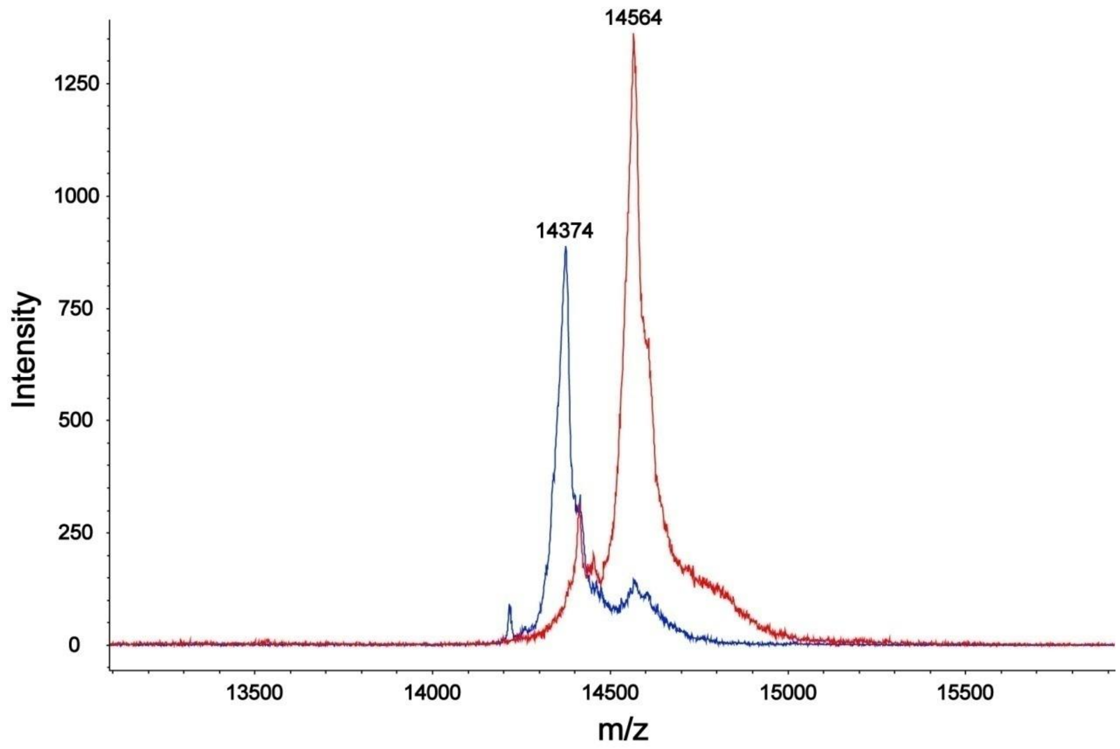

Figure S9. The ligation reaction of MTSL with DgkA mutants was examined by matrix-assisted laser desorption/ ionization time of flight mass spectrometry (MALDI-TOF-MS) shown here for 46C-DgkA (blue) and spin-labeled 46C-DgkA (red) samples. The molecular weight of the spin-labeled 46C-DgkA is ~190 Da higher than that of unlabeled 46C-DgkA, which is similar to the theoretical value of 185 Da. Similar experiments were performed to monitor the extent of completion of labeling reactions on all the other single cysteine mutant sites.

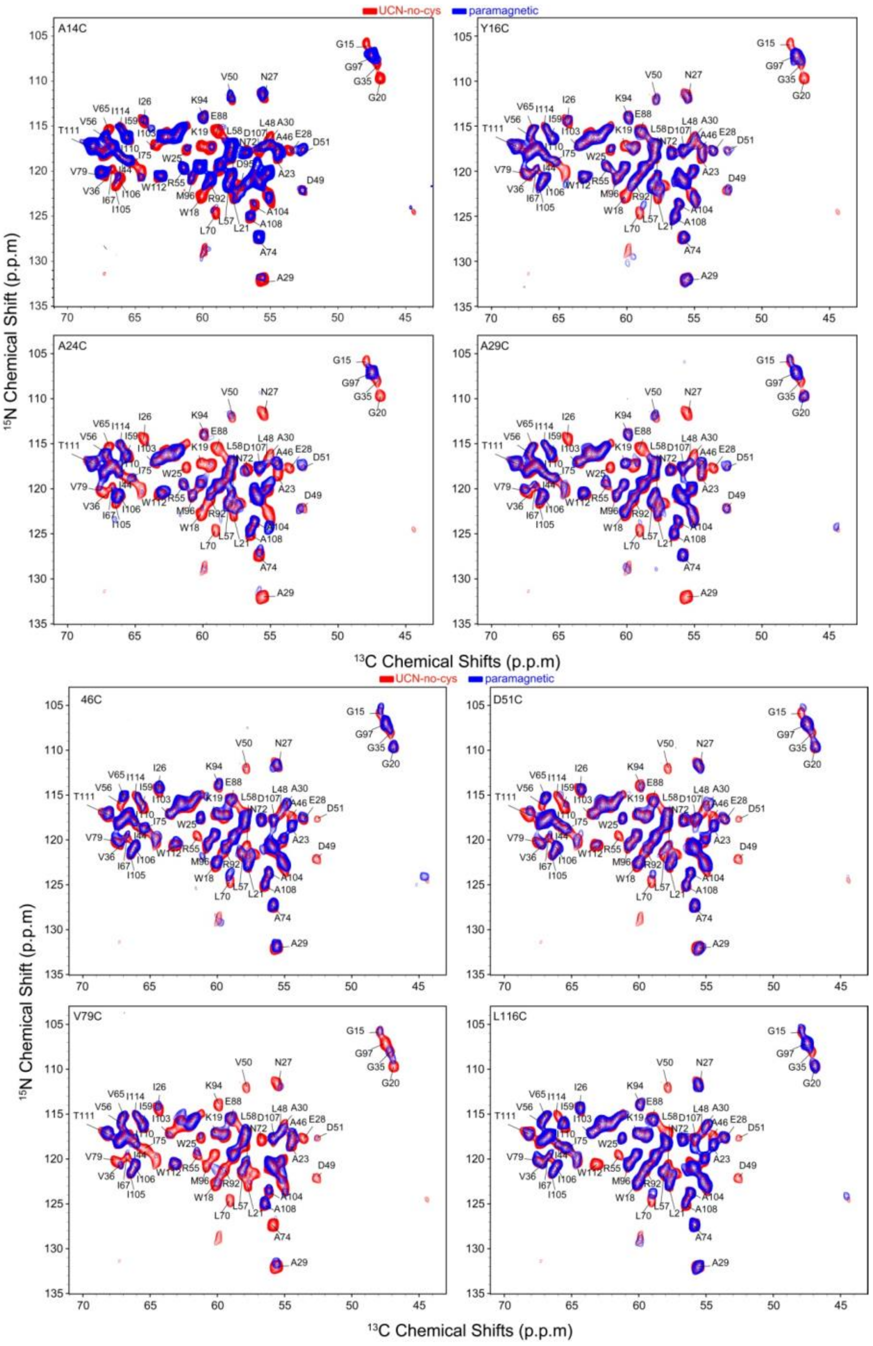

Figure S10. Comparisons of 2D NCA spectra of each used MTSL-labeled mutant samples (blue) with no-cys ones (red) showing the correct folding of DgkA in the mutant samples.

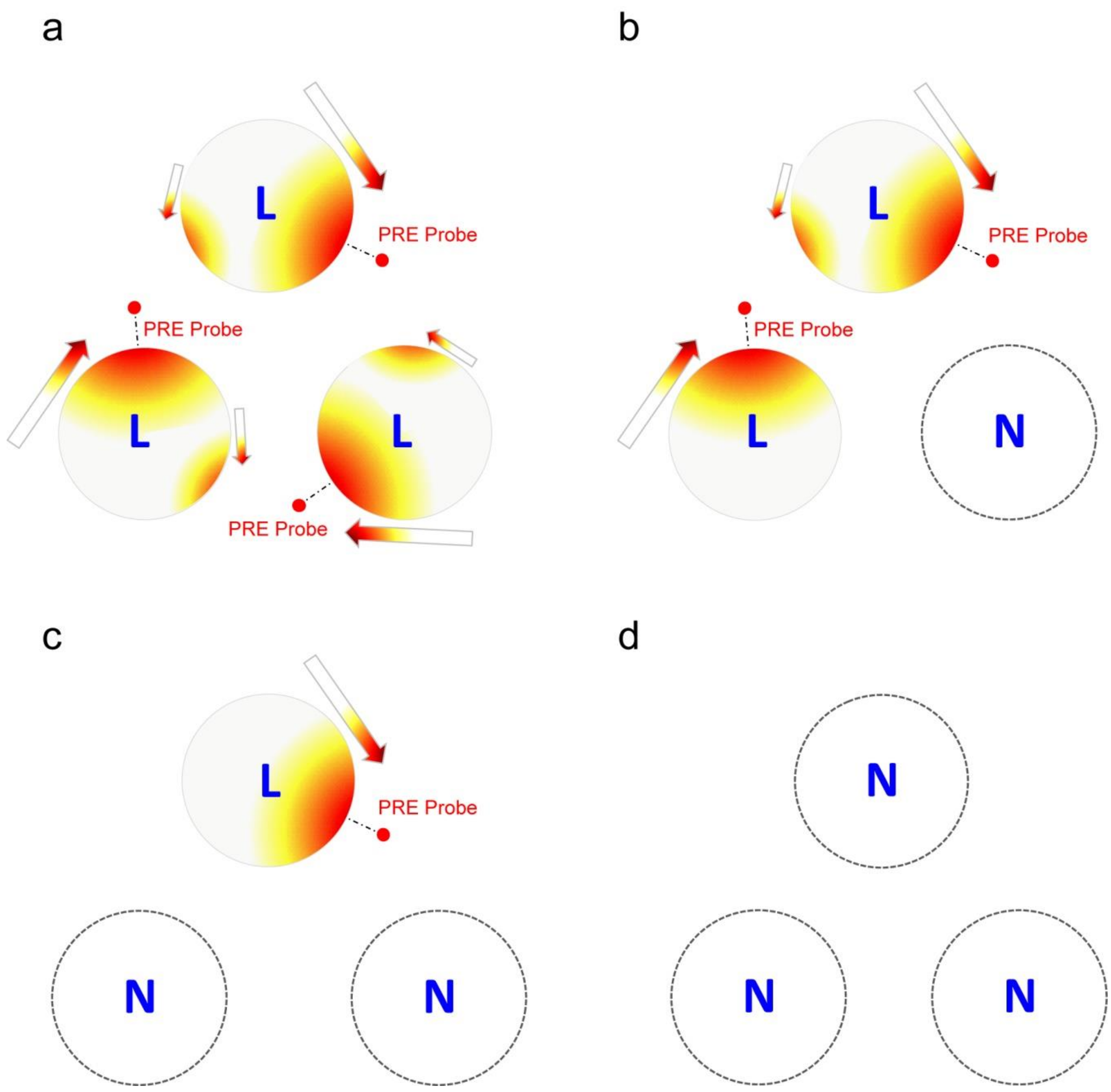

Figure S11. Four possible schemes of dilution of MTSL-U-^{13}C, ^{15}N-DgkA by natural abundance DgkA with MTSL-U-^{13}C, ^{15}N-DgkA : na-no-cys-DgkA = 1:4 for the observation of intra-monomer PREs. 'L' represents uniform ^{13}C,^{15}N isotope labeled DgkA monomer and 'N' is the natural abundance no-cys DgkA monomer. PRE probes are highlighted as red balls. By a random combination of different monomers, the probability of occurrence for each scheme represented by a, b, c, and d is 0.8, 9.6, 38.4, and 51.2%, respectively. The probability is calculated as follows:

$P_a = (1/5)^3 = 1/125$

$P_b = 1/5\times1/5\times4/5\times C_3^1 = 12/125$

$P_c= 1/5\times4/5\times4/5\times C_3^1 = 48/125;$

$P_d = (4/5)^3 = 64/125$

1) If the PRE probe influences the signal of only one other monomer (as shown in the b scheme), the probability P of uniform ^{13}C,^{15}N isotope labeled DgkA monomers affected by inter-monomer PREs is

$$P = (3\times1/125+12/125)/(3/125+12/125+12/125+48/125) = 15/75 = 20\%.$$

2) If the PRE probe impacts signal of other two monomers in the b scheme, the probability P of uniform ^{13}C,^{15}N isotope labeled DgkA monomers affected by inter-monomer PREs is

$$P = (3\times1/125+2\times12/125)/(3/125+12/125+12/125+48/125) = 27/75 = 36\%.$$

This possibility is only for the spin-labeled site situated at the core of the trimer.

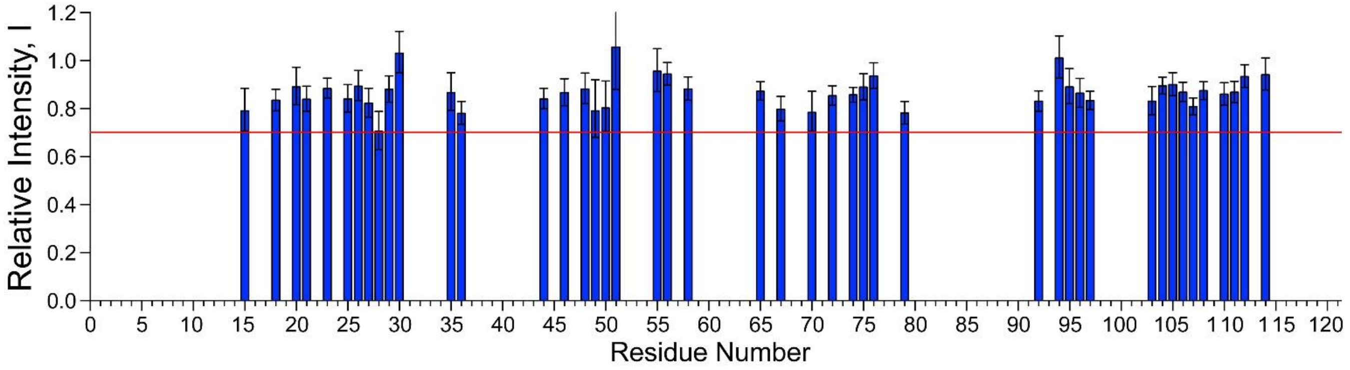

Figure S12. Stability analysis of relative intensity of individual cross peaks in the 2D NCA spectra of U-^{13}C, ^{15}N-no-cys-DgkA. The 2D NCA experiments were performed twice using the same sample and same experimental parameters. In the plot, relative intensity I is defined as $I = (I_1/I_2)/(I_1/I_2)max$, where I_1 and I_2 are the heights of individual cross peaks in the two replicated NCA spectra. Relative intensities of the residues without resolved cross peaks were set to zero.

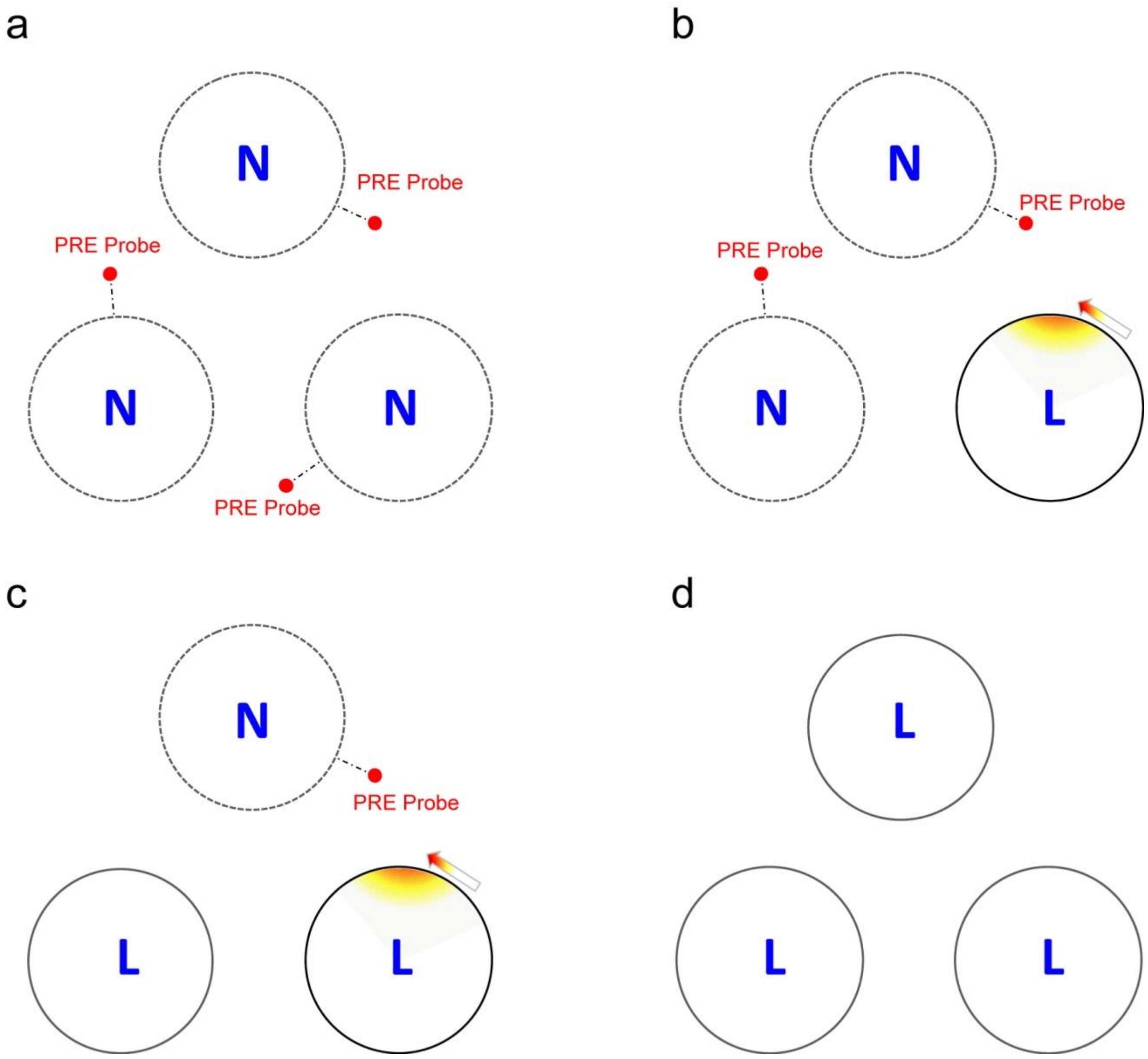

Figure S13. Four possible schemes of the mixing labeled sample MTSL-na-DgkA : U-^{13}C, ^{15}N-no-cys-DgkA = 2:1 for the observation of inter-monomer PREs. 'L' and 'N' represents uniformly ^{13}C,^{15}N labeled and natural abundance no-cys DgkA monomers, respectively. PRE probes are highlighted as red balls. By a random combination of different monomers, the probability of occurrence for each scheme a, b, c, and d was calculated as follows:

$P_a = (2/3)^3 = 8/27 = 29.6\%$

$P_b = 1/3 \times 2/3 \times 2/3 \times C_3^1 = 12/27 = 44.5\%$

$P_c = 1/3 \times 1/3 \times 2/3 \times C_3^1 = 6/27 = 22.2\%$

$P_d = (1/3)^3 = 1/27 = 3.7\%$

1) If the PRE probe only impact NMR signal of one other monomer (as shown in the scheme c), the probability P of uniform ^{13}C,^{15}N isotope labeled DgkA monomers would not be affected by inter-monomer PREs at all, is:

$$P = (6/27 + 3\times 1/27)/(6/27+3/27+12/27+6/27) = 9/27 = 33.3\%.$$

2) If the PRE probe impacts NMR signal from two other monomers in the scheme c, the probability P of uniformly ^{13}C,^{15}N labeled DgkA monomers not being affected by inter-monomer PREs at all, is:

$$P = (3\times 1/27)/(6/27+3/27+12/27+6/27) = 9/27 = 11.1\%.$$

This possibility is only for the spin-labeled site located at the core of the trimer.

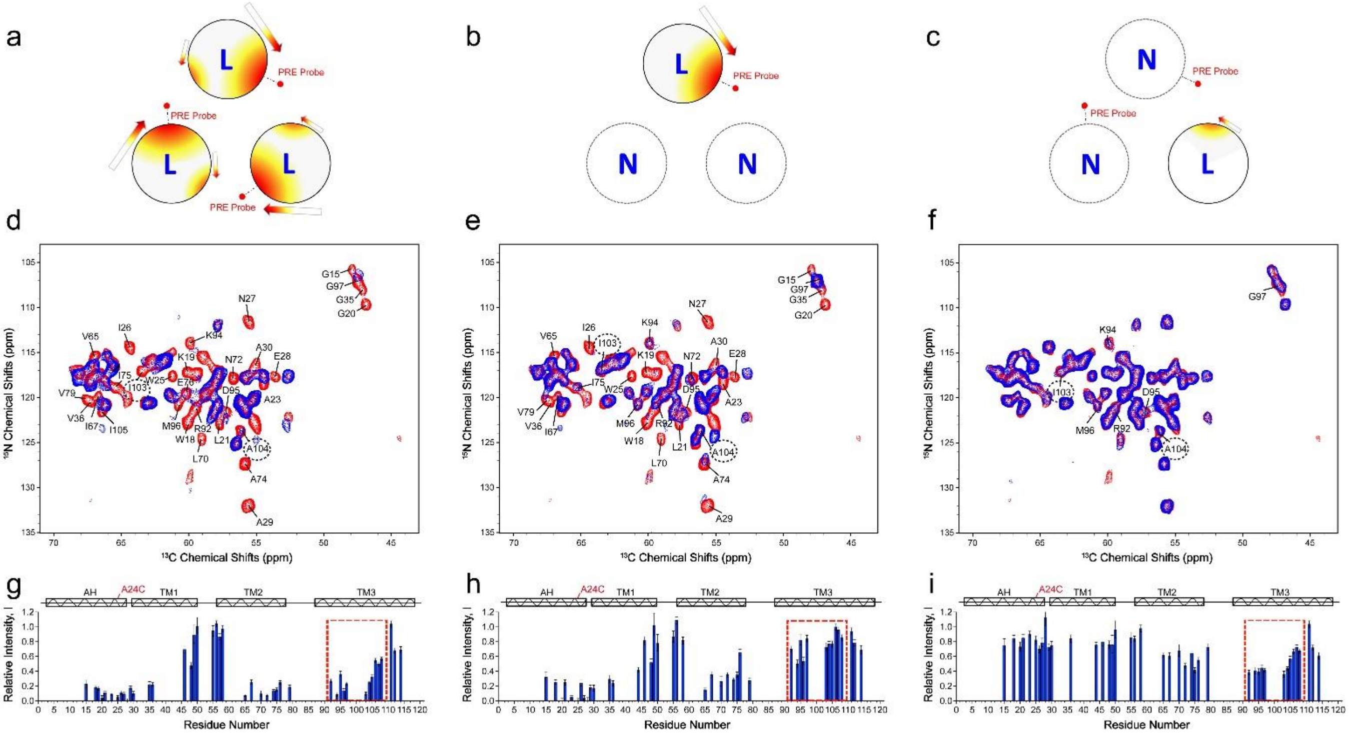

Figure S14. Observation of intra- and inter-monomer PREs by using different labeling strategies. (a-c) Schematic of DgkA trimer with various isotope and spin-labeling schemes. 'L' and 'N' represent uniformly ^{13}C,^{15}N labeled and natural abundance DgkA monomers, respectively. PRE probes are highlighted as red balls. (d-f) 800 MHz 2D NCA ssNMR spectra of A24C-DgkA samples with different labeled schemes corresponding to a, b, and c, respectively (blue), superimposed on that of the spectra of diamagnetic (control) no-cys-DgkA samples (red). Residues strongly affected by PREs are labeled. (g-i) Site-specific comparison of relative intensity of resolved cross peaks in NCA spectra corresponding to d, e, and f, respectively. Considering the possible differences in the amount of various samples, the relative intensity is defined as $I = (I_{para}/I_{dia})/(I_{para}/I_{dia})_{max}$, where I_{para} and I_{dia} are the peak heights in NCA spectra of paramagnetic A24C and diamagnetic control no-cys-DgkA samples, respectively, and $(I_{para}/I_{dia})_{max}$ is the maximum value of I_{para}/I_{dia}. The noise levels of the corresponding peaks were shown by error bars. The secondary structure of DgkA with spin labeling at the A24C residue is shown on the top. Residues affected by inter-monomer PREs of A24C are indicated by dashed rectangles.

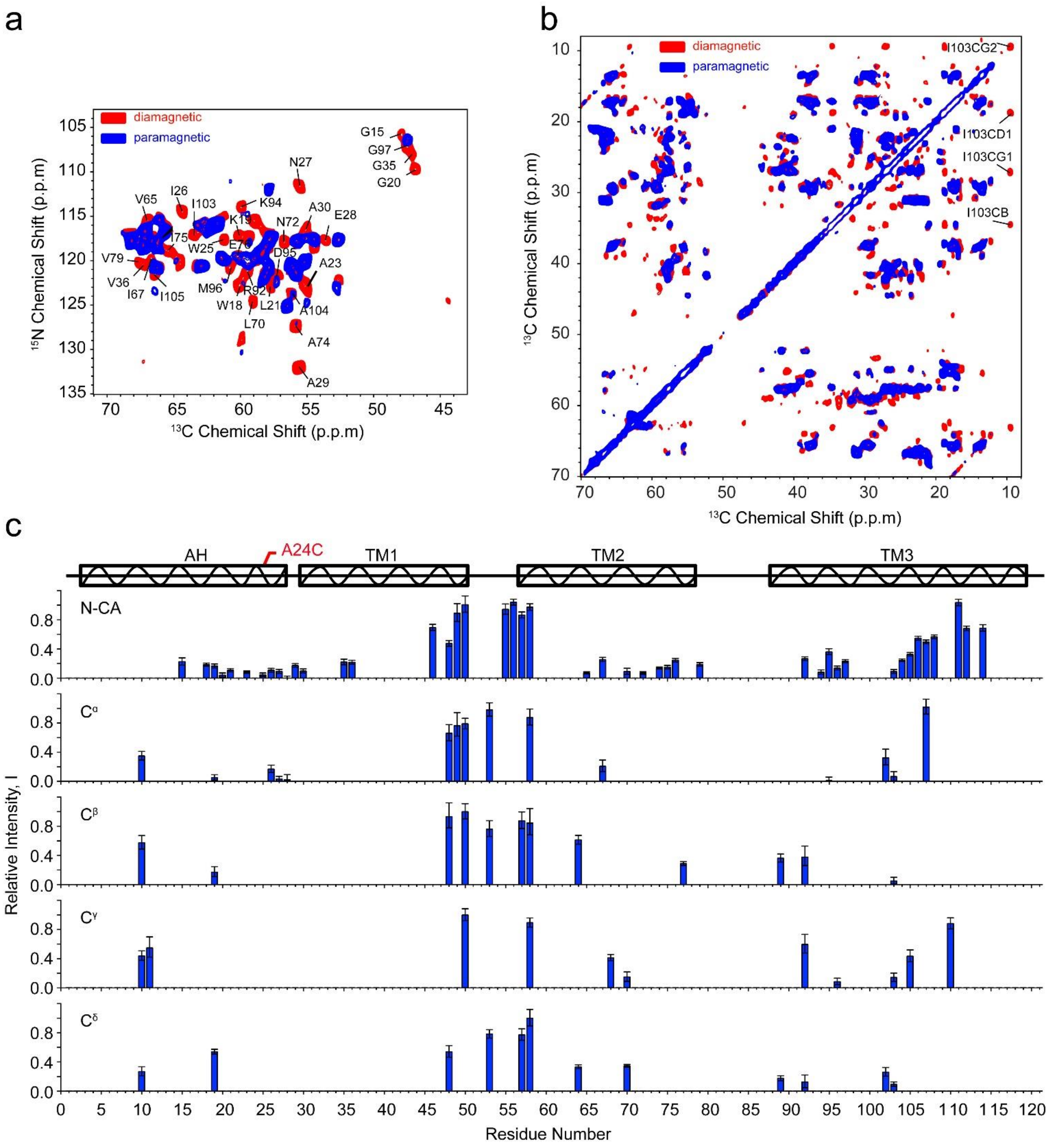

Figure S15. PRE induced intensity reduction of the resolved cross peaks in 2D NCA and DARR (mixing time = 50 ms) spectra, respectively. 800 MHz ssNMR spectra of 2D NCA (a) and DARR (b) of MTSL labeled U-^{13}C, ^{15}N-A24C-DgkA sample (blue) and the spectra of diamagnetic control U-^{13}C, ^{15}N -no-cys-DgkA sample (red). The residues strongly affected by PREs are labeled. (c) The site-specific comparison of relative intensities of cross peaks in 2D NCA and ^{13}C-^{13}C DARR spectra, corresponding to C_α, C_β, C_γ and C_δ resonances as the direct dimension, respectively. The relative intensity I is defined as $I = (I_{para}/I_{dia})/(I_{para}/I_{dia})_{max}$.

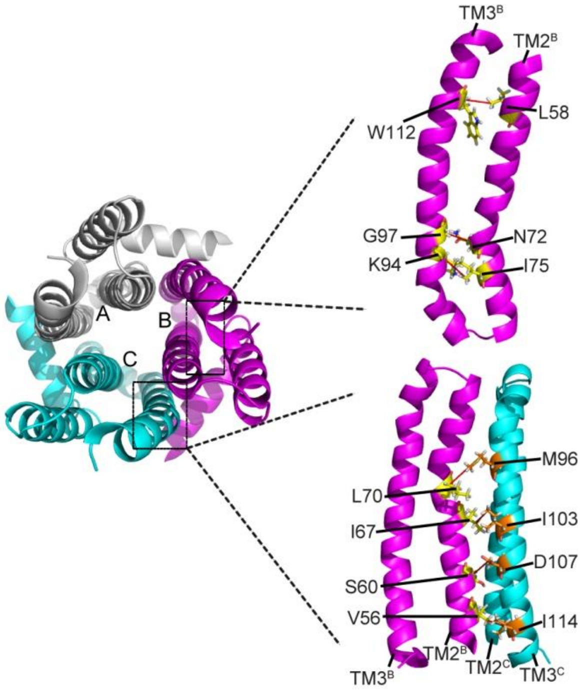

Figure S16. Mapping of long-range (|i-j|>5) intra-monomer (upper inset) and inter-monomer (lower inset) contacts collected from the 2D ^{13}C-^{13}C DARR ssNMR spectra to the lowest energy DgkA structure. Monomers A, B, and C are colored in gray, magenta and cyan, respectively. A zoomed-in structure of TM2 and TM3 of monomer B is displayed (upper inset) with three measured intra-monomer ^{13}C-^{13}C distances (red lines). In addition, four inter-monomer ^{13}C-^{13}C distances between TM2/TM3 of monomer B and TM3 of monomer C are displayed in the zoomed structure (lower inset).

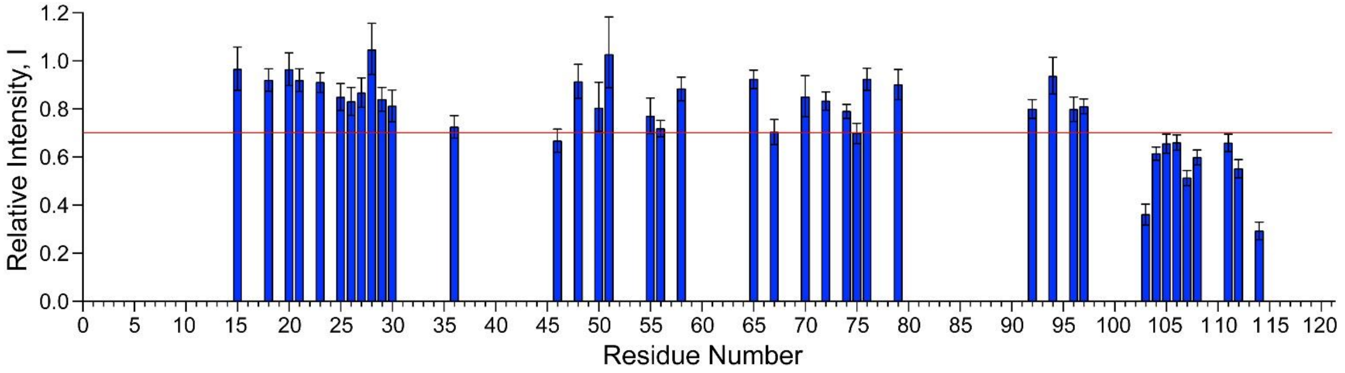

Figure S17. Site-specific comparison of the relative intensity of resolved cross peaks in the NCA spectra corresponding to the 46C site for observing inter-monomer PREs. The relative intensity is defined as $I = (I_{para}/I_{dia})/(I_{para}/I_{dia})_{max}$, where I_{para} and I_{dia} are the peak heights in NCA spectra of the paramagnetic 46C DgkA and diamagnetic control no-cys-DgkA samples, respectively. $(I_{para}/I_{dia})_{max}$ is the maximum value of I_{para}/I_{dia}. The red line indicates the I value of 0.7.

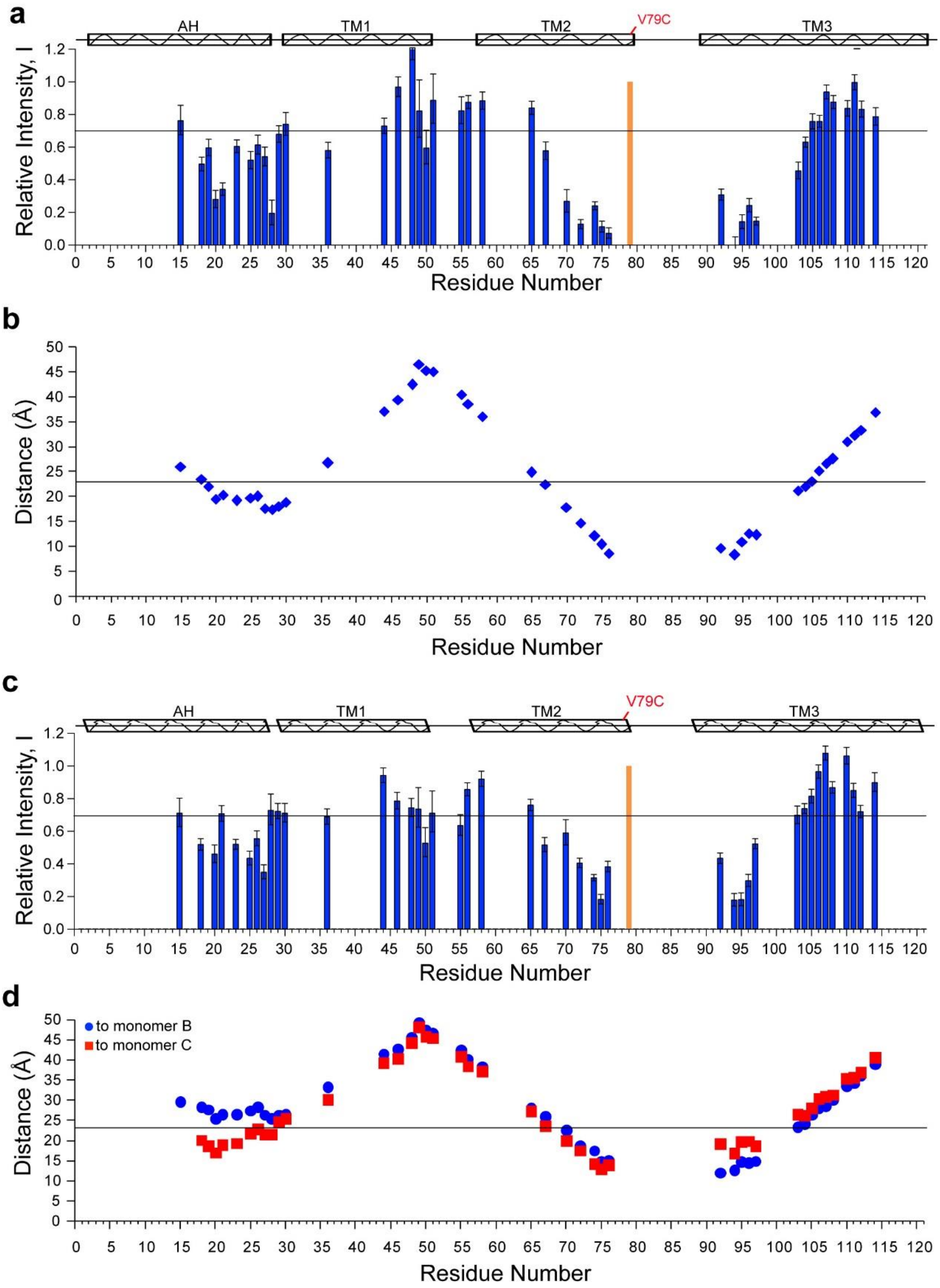

Figure S18. Correlations of experimentally measured relative intensities of the peaks in the NCA spectra with intra-(a) and inter monomer PREs (c) to the corresponding distances in the X-ray structure of DgkA(b)(d). The lines in (a)(c) and (b)(d) indicate the relative intensities of 0.7 and the corresponding distances of 23 Å, respectively.

		1	10	20	30 40
wt-DgkA	MGHHHHHHEL	ANNTTGFTRI	IKAAGYSWKG	LRAAWINEAA	FRQEGVAVLL
no-cys-DgkA	MGHHHHHHEL	ANNTTGFTRI	IKAAGYSWKG	LRAAWINEAA	FRQEGVAVLL

	50	60	70	80	90
wt-DgkA	AVVIACWLDV	DAITRVLLIS	SVMLVMIVEI	LNSAIEAVVD	RIGSEYHELS
no-cys-DgkA	AVVIAAWLDV	DAITRVLLIS	SVMLVMIVEL	LNSAIEAVVD	RIGSEYHELS

	100	110	120	
wt-DgkA	GRAKDMGSAA	VLIAIIVAVI	TWCILLWSHF	G
no-cys-DgkA	GRAKDMGSAA	VLIAIIDAVI	TWAILLWSHF	G

Figure S19. Amino acid sequence of the WT and no-cys-DgkA mutants used in this work.

Table S1. Distance range from intra-monomer PREs

Distance (Å)	Relative Intensity I	Sample
d<15 (attractive)	I < 0.33	diluted
11<d<27 (medium)	0.33 < I < 0.70	diluted
d>23 (repulsive)	I > 0.7	undiluted or diluted

Table S2. Samples for extracting PRE contacts

Sites	Intra-PRE	Inter-PRE	Undiluted
A14C	-	-	+
Y16C	+	-	+
A24C	+	+	+
A29C	+	-	-
46C	+	+	-
D51C	+	-	-
V79C	+	+	-
L116C	+	-	-

"+" or "-" represent samples which were used or not be used, respectively.

Table S3. Distance range from inter-monomer PREs in the first step of structure calculation

Distance (Å)	Relative Intensity I	Samples	Possibility of monomers
d>23	I > 0.7	undiluted or diluted	A-B, A-C or A-B&C
d<23	I < 0.7	Undiluted or diluted	A-B, A-C or A-B&C

Table S4. Distance range from inter-monomer PREs in the second step of structure calculation

Distance (Å)	Relative Intensity I	Samples
d<15	I < 0.33	undiluted
11<d<27	0.33 < I < 0.70	undiluted
d>23	I > 0.7	undiluted or diluted
d<23	I < 0.7	diluted

Table S5. Comparison of ^{13}C-^{13}C distance restraints with those in the 10 lowest-energy structures calculated from PRE-CS-Rosetta

atom1	atom2	distances from ssNMR structures	distances from DARR	Inter/intra-monomer
W112CZ2	L58CA	6.6 ± 2.2	5.5 ± 3.0	a
W112CZ2	L58CB	6.5 ± 1.7	5.5 ± 3.0	a
W112CZ2	L58CD1	6.8± 1.2	5.5 ± 3.0	a
W112CD2	L58CA	6.6± 1.5	5.5 ± 3.0	a
W112CH2	L58CA	7.6 ± 2.2	5.5 ± 3.0	a
A108 CA	S61CA	6.7 ± 0.5	5.0 ± 2.5	a
W112CZ2	L58CG	6.3 ± 1.4	5.0 ± 2.5	a
W112CH2	L58CD2	6.2± 1.4	5.0 ± 2.5	a
W112CH2	L58CG	7.1 ± 1.5	5.0 ± 2.5	a
W112CZ2	L58CD2	5.4 ± 1.4	5.0 ± 2.5	a
W112CE2	L58CD2	4.7 ± 1.1	5.0 ± 2.5	a
W112CA	L58CD2	5.4 ± 1.1	5.0 ± 2.5	a
K94 CB	I75CD1	5.4 ± 1.5	5.0 ± 2.5	a
K94 CB	I75CG2	5.6 ± 1.2	5.0 ± 2.5	a
K94 CG	I75CA	7.1 ± 1.1	5.0 ± 2.5	a
G97 CA	N72CB	5.5 ± 0.5	5.0 ± 2.5	a
V56CG1	I110CB	8.8 ± 0.5	5.0 ± 2.5	b
V56CG2	I110CB	8.4 ± 0.6	5.0 ± 2.5	b
V56CG1	I114CB	6.5± 0.9	5.0 ± 2.5	b
V56CG2	I114CB	4.5 ± 1.1	5.0 ± 2.5	b
S60CA	D107CG	6.5 ± 0.8	5.0 ± 2.5	b
I67CB	I103CB	6.4 ± 0.7	5.0 ± 2.5	b
I67CB	I103CD1	7.0 ± 1.0	5.0 ± 2.5	b
I67CG1	I103CD1	6.1 ± 1.0	5.0 ± 2.5	b
L70CA	M96CG	6.2 ± 1.1	5.0 ± 2.5	b
L70CD1	M96CG	6.7 ± 0.5	5.0 ± 2.5	b

1. The red color highlights the 4 distance restraints of the intra-monomer assigned from the diluted sample. The others were distinguished from the structures calculated from PRE-CS-Rosetta.
2. a – intra-monomer; b – inter-monomer
