# Supplementary material for: Structure of membrane diacylglycerol kinase in lipid bilayers
Source: Commun Biol. 2021 Mar 5;4:282. doi: 10.1038/s42003-021-01802-1 (PMC7935881; doi:10.1038/s42003-021-01802-1)
Supplement: Supplementary file 2 — Description of Additional Supplementary Files. [file 42003_2021_1802_MOESM2_ESM.pdf]

Description of additional supplementary files

File name: Supplementary Data 1

Description: Chemical shifts of no-cys-DgkA reconstituted in *E. coli* membrane extracts

File name: Supplementary Data 2

Description: Source data for Figures 3d and 4c
